# Landscape of genomic alterations in high-grade serous ovarian cancer from exceptional long- and short-term survivors

**DOI:** 10.1186/s13073-018-0590-x

**Published:** 2018-10-31

**Authors:** S. Y. Cindy Yang, Stephanie Lheureux, Katherine Karakasis, Julia V. Burnier, Jeffery P. Bruce, Derek L. Clouthier, Arnavaz Danesh, Rene Quevedo, Mark Dowar, Youstina Hanna, Tiantian Li, Lin Lu, Wei Xu, Blaise A. Clarke, Pamela S. Ohashi, Patricia A. Shaw, Trevor J. Pugh, Amit M. Oza

**Affiliations:** 1 Princess Margaret Cancer Centre, University Health Network, 610 University Avenue, Toronto, Ontario M5G 2M9 Canada; 2 Department of Medical Biophysics, University of Toronto, Toronto, Ontario Canada; 3 Department of Medicine, University of Toronto, Toronto, Canada; 4 Department of Laboratory Medicine and Pathobiology, University of Toronto, Toronto, Canada; 5 Department of Pathology, University Health Network, Toronto, Canada; 6 Department of Immunology, University of Toronto, Toronto, Canada; 7 Ontario Institute for Cancer Research, Toronto, Canada

**Keywords:** Ovarian cancer, Immuno-genomics, Tumor microenvironment

## Abstract

**Background:**

Patients diagnosed with high-grade serous ovarian cancer (HGSOC) who received initial debulking surgery followed by platinum-based chemotherapy can experience highly variable clinical responses. A small percentage of women experience exceptional long-term survival (long term (LT), 10+ years), while others develop primary resistance to therapy and succumb to disease in less than 2 years (short term (ST)). To improve clinical management of HGSOC, there is a need to better characterize clinical and molecular profiles to identify factors that underpin these disparate survival responses.

**Methods:**

To identify clinical and tumor molecular biomarkers associated with exceptional clinical response or resistance, we conducted an integrated clinical, exome, and transcriptome analysis of 41 primary tumors from LT (*n* = 20) and ST (*n* = 21) HGSOC patients.

**Results:**

Younger age at diagnosis, no residual disease post debulking surgery and low CA125 levels following surgery and chemotherapy were clinical characteristics of LT. Tumors from LT survivors had increased somatic mutation burden (median 1.62 vs. 1.22 non-synonymous mutations/Mbp), frequent BRCA1/2 biallelic inactivation through mutation and loss of heterozygosity, and enrichment of activated CD4+, CD8+ T cells, and effector memory CD4+ T cells. Characteristics of ST survival included focal copy number gain of *CCNE1*, lack of *BRCA* mutation signature, low homologous recombination deficiency scores, and the presence of *ESR1-CCDC170* gene fusion.

**Conclusions:**

Our findings suggest that exceptional long- or short-term survival is determined by a concert of clinical, molecular, and microenvironment factors.

**Electronic supplementary material:**

The online version of this article (10.1186/s13073-018-0590-x) contains supplementary material, which is available to authorized users.

## Background

High-grade serous ovarian cancer (HGSOC) is the most lethal gynecologic malignancy, accounting for 70–80% of ovarian cancer deaths worldwide [1]. Despite promising results with cytoreductive surgery and platinum-based chemotherapy, more than 75% of women with HGSOC will relapse after completion of first-line therapy [2]. The window of opportunity to tailor therapeutic interventions to control progressive disease is limited due to the inherent cellular heterogeneity and genomic instability of HGSOC. While platinum chemotherapy is the cornerstone of contemporary treatment, ultimately, the majority of women with epithelial ovarian cancer (EOC) will develop chemotherapy resistance and succumb to their disease within 5 years of diagnosis (46.2% 5-year survival) [3]. However, 16% of patients with serous histology experience overall survival greater than 10 years [4]. In contrast, other patients diagnosed at the same disease stage and treated with similar therapeutic approaches will experience rapid disease progression. Current clinical algorithms cannot discern these patient survival outcomes at the time of diagnosis and therefore patients are given similar treatment.

In many ovarian cancer studies, age at diagnosis, disease stage, grade, histology, residual disease post-surgery, and disease recurrence have been identified and validated to have prognostic value [4, 5]. Molecular characteristics such as *BRCA1/2* mutations [6, 7] and homologous repair deficiency in HGSOC have been demonstrated and validated as predictive of response to platinum therapy and poly-ADP polymerase (PARP) inhibitors [7–9]. In addition, recent publications have demonstrated that immune cell populations infiltrating ovarian tumor tissue may be prognostic [10–14]. However, without complete long-term follow-up information to accompany patient and tumor molecular profiles, clinical and molecular factors that contribute to long-term (LT) and short-term (ST) survival in HGSOC remain elusive.

In this pilot study, we sought to identify clinical and molecular factors that distinguish HGSOC patients who share similar clinical characteristics and pathology at diagnosis with exceptional survival outcomes, either LT or ST, through integrated analysis of clinical features, germline variants, somatic genomic alterations, and tumor immune microenvironment.

## Methods

### Sample inclusion criteria

We identified patients from the Princess Margaret Cancer Registry diagnosed with HGSOC who underwent primary debulking surgery. To obtain a clinically homogeneous population at diagnosis, we selected patients with the following criteria: (1) diagnosis of advanced HGSOC confirmed by an expert gynecologic pathologist and stage III according to the FIGO classification; (2) primary debulking surgery followed by at least 6 cycles of platinum-based chemotherapy; and (3) availability of chemotherapy-naïve tumor and matched normal tissue of sufficient quantity and quality for molecular analysis. Patient cohorts representing extreme tails of the HGSOC overall survival distribution were selected for comparison in this study. Short-term survival patients were defined as patients with (1) overall survival between 6 months and 2 years, (2) primary platinum resistance, and (3) documented disease progression within 6 months from completing platinum-based chemotherapy. Patients with LT survival had durable platinum sensitivity and were identified based on OS greater than 10 years following HGSOC diagnosis (Additional file 1: Figures S1, S2A). The presence of residual disease post debulking surgery was collected from the original surgical notes.

### Patient tissues processing

Treatment-naïve frozen or formalin-fixed paraffin-embedded (FFPE) preserved primary HGSOC tumors and matched normal tissues from these patients were obtained from the University Health Network Biobank with Research Ethics Board approval. DNA and RNA were co-isolated from available tissues using Qiagen AllPrep DNA/RNA/miRNA Universal kit or the Qiagen AllPrep DNA/RNA FFPE kit following the manufacturer’s protocol.

### TCGA data

TCGA data for HGSOC was downloaded from Broad GDAC Firebrowse (http://firebrowse.org/?cohort=OV/). RNA-seq V2 FASTQ files for each TCGA OV sample was downloaded from Genomic Data Commons Data Portal (https://portal.gdc.cancer.gov).

### Exome and RNA sequencing

Exome libraries were constructed from 200ng starting genomic DNA using the Agilent SureSelect Human All Exon V5+UTRs kit. One hundred base pair paired-end reads were sequenced using Illumina HiSeq 2000 or 2500 instruments to 250X target read depth for tumor and 50X for normal tissue libraries. Tumor RNA libraries were prepared from 200ng of RNA using the Illumina TruSeq Stranded Total RNA kit with Ribo-Zero Gold. Libraries were sequenced with pair-end 100 cycles V3 using Illumina HiSeq 2000 to achieve a minimum of ~ 80 million reads per sample. Whole exome FASTQ files were aligned to reference human genome hg19 using BWA [15] and pre-processed following GATK Best Practices Protocol [16, 17]. RNA-seq FASTQ files were aligned to human genome version hg19 and transcript annotation GENCODE v19 (Additional file 2).

### Mutational profiling

Germline variants were called using GATK HaplotypeCaller (version 1.130) from normal tissue BAM files with default settings. Somatic mutations were called from tumor/normal BAM file pairs using muTect (version 1.1.4) [18], Varscan2 (version 2.4.2) [19], and Strelka (version 1.0.14)) [20] for single nucleotide variations (SNVs) and small insertions and deletions (Indels) on paired normal and tumor tissue BAM files. Mutations were annotated using Oncotator (version 1.5.3) [21]. Deep sequencing of all coding exons of *TP53* was performed on all tumors lacking detectable *TP53* mutation in exome data using custom hybrid-capture probes (Additional file 2).

### CNV profiling

Sequencing depth ratios for each tumor and normal exome pair were collected using GATK mpileup (version 3.3.0) using paired sample mode. Varscan2 (version 2.4.2) [19] was used to identify contiguous segments of DNA with similar depth ratio and variant allele frequencies. Given DNA copy segments and SNPs, and tumor cellularity estimate from *TP53* mutation allele fraction, Sequenza (version 2.1.2) [22] was used to estimate the tumor ploidy and allele-specific copy number for each DNA segment. GISTIC2 (version 2.0.22) [23] was used to identify recurrent somatic copy number alterations (SCNAs) across the cohort and within each survival group. For copy number analysis of specific genes such as *TP53*, *BRCA1*, *BRCA2*, and *CCNE1*, segment files containing total and allele-specific copy numbers were annotated using a custom R script. We defined a focally amplified gene (defined as < 3 Mb according to Krijgsman et al. [24]) as having a copy number greater than the estimated sample ploidy plus 2. We selected a purity-corrected absolute copy number of 2 above background ploidy (i.e., ploidy = 4 for largely diploid genomes) as this is the threshold commonly used for reporting clinical cytogenetic alterations in cancer. We also selected this relatively high threshold to avoid reporting false-positive variants from arm-level chromosomal alterations inherent to the highly complex genomes found in ovarian cancer, as well as the varying tumor content levels encountered in clinical specimens such as those used in our study. As shown in Additional file 3, this approach ensures that we are focused on clearly focally amplified regions that stand out from a highly aneuploid background. Loss of heterozygosity (LOH) was defined as the lack of the alternate allele (B allele copy number = 0). A focal gene deletion was defined as copy number less than the global ploidy minus 1 and lacking the alternate allele. The HRD-LOH score, the number of large (> 15 Mbp, less than a chromosome arm) LOH genomic segments, was determined for each tumor CNV profile.

### Immune enrichment analysis

We used single sample gene set enrichment analysis (ssGSEA) [25] to assess the gene set activation score of each tumor specimen (LT (*n* = 13), ST (*n* = 16)). Immune-reactive HGSOC subtype [26] and ESTIMATE immune score [27] gene sets were used to infer overall immune infiltration by ssGSEA. Gene sets describing specific immune cell types (activated CD8^+^ T, activated CD4^+^ T, T cells, effector memory CD8^+^ T, effector memory CD4^+^ T, NK cells, macrophages, T-regs, and activated B cells) are used to infer cell-type-specific infiltration levels [28]. GSVA R-package (version 1.22) [29] implementation of ssGSEA was used to calculate sample scores. For each gene set, *z*-score normalization of ssGSEA scores centered at medians was applied across all samples.

### Fusion gene detection

Tophat fusion (tophat2 version 2.0.8b) [30] with default parameters was used to nominate potential fusion transcripts from RNA-seq data. Fusion candidates were filtered and prioritized based on total number of junction spanning reads (> 10), read pairs spanning fusion gene partners (> 2), and read pairs containing a read that partially span the fusion junction (> 0).

### Statistical methods

To compare continuous variables such as mutation frequency, gene-expression, HDR-LOH score, and gene-set enrichment scores between two groups, two-sided non-parametric Wilcoxon Rank Sum tests were used to assess statistical significance. Two-sided Fisher’s exact tests were used for comparisons of discrete or dichotomized variables such as *BRCA* mutation enrichment, *TP53* mutation enrichment, *CCNE1* amplification enrichment, HRD-LOH scores, and HRD mutation signature enrichment. Given two categorical variables, Fisher’s exact test was applied to assess whether the proportions of one categorical variable are independent of the other one. Wilcoxon Rank Sum tests were conducted to test whether the medians of the distributions of a continuous variable in stratified groups are the same. Spearman correlation was conducted to test the monotonic relationship between two continuous variables. Two-sided tests were conducted with significance level at 0.05. All data consolidation, statistical testing, and data visualization were performed using SAS 9.4 and R-scripts in the R (version 3.3.1) [31] statistical environment. Power analysis is provided in Additional file 2.

## Results

### Clinical description of the study cohort

From 829 patients with HGSOC entered in the Princess Margaret (PM) Cancer Registry from 2000 to 2013, we selected two cohorts of patients with exceptionally ST (< 2 years, 20 patients) and LT OS (≥ 10 years, 21 patients) (Table 1, Additional file 1: Figures S1, S2A). On average, patients with LT survival were younger than ST (56 vs. 61 years mean age at diagnosis) and were less likely to have residual disease post-surgery (35% versus 76%). Disease recurred in all ST patients and 3 (3/20, 15%) LT patients. Cancer antigen 125 (CA125) levels in the blood serum at diagnosis did not correlate with survival; however, LT survivors had significantly lower CA125 levels post-surgery and at the end of chemotherapy (Table 1) (*p* <  0.001).

**Table 1:** Clinical characteristics of patients diagnosed with stage III, grade III, serous ovarian epithelial cancer at Princess Margaret by length of survival

Covariate	Full Sample (*n* = 41)	LT (*n* = 20)	ST (*n* = 21)	*p* value
**Number of patients**	41	20	21	
**Stage III, HGSOC**	41 (100)	20 (49)	21 (51)	
**Overall Survival**				*< 0.001*
< 6 months	0 (0)	0 (0)	0 (0)	
6–12 months	2 (5)	0 (0)	4 (19)	
12–24 months	19 (46)	0 (0)	17 (81)	
> 24 months	20 (49)	20 (100)	0 (0)	
**Age at diagnosis**				*0.024*
Mean (sd)	59 (9.3)	56.1 (9.4)	61.7 (8.7)	*0.024*
Median (min,max)	57 (40,84)	55.5 (40,84)	59 (47,76)	
**Residual disease**				*0.012*
No	18 (44)	13 (65)	5 (24)	
Yes	23 (56)	7 (35)	16 (76)	
**Disease recurrence**				*< 0.001*
No	17 (41)	17 (85)	0 (0)	
Yes	24 (59)	3 (15)	21 (100)	
**Number of disease recurrence**				*< 0.001*
0	17 (41)	17 (85)	0 (0)	
1	15 (37)	1 (5)	14 (67)	
2	7 (17)	1 (5)	6 (29)	
> 2	2 (5)	1 (5)	1 (5)	
**CA125 at diagnosis**				0.39
Mean (sd)	1207 (1781.6)	870.4 (863.1)	1491 (2277.9)	
Median (min,max)	475 (67,9162)	585 (67,2700)	399 (184,9162)	
Missing	**6**	**4**	**2**	
**CA125 at diagnosis rate**				0.41
Unknown	6 (15)	4 (20)	2 (10)	
0–35 U/mL	0 (0)	0 (0)	0 (0)	
> 35 U/mL	35 (85)	16 (80)	19 (90)	
**CA125 post-surgery**				*< 0.001*
Mean (sd)	421 (932.4)	63.9 (74.8)	799.1 (1243.4)	
Median (min,max)	121 (7,4712)	33 (7299)	296 (53,4712)	
Missing	**6**	**2**	**4**	
**CA125 post-surgery rate**				*< 0.001*
Unknown	6 (15)	2 (10)	4 (19)	
0–35 U/mL	9 (22)	9 (45)	0 (0)	
> 35 U/mL	26 (63)	9 (45)	17 (81)	
**CA125 post chemotherapy**				*< 0.001*
Mean (sd)	656.4 (3772.9)	4.6 (2.1)	1308 (5325.7)	
Median (min,max)	6.5 (2,23,290)	4 (2,10)	18 (4, 23,287)	
Missing	**3**	**1**	**2**	
**CA125 post chemotherapy rate**				*0.0063*
Unknown	3 (7)	1 (5)	2 (10)	
0–35 U/mL	31 (76)	19 (95)	12 (57)	
> 35 U/mL	7 (17)	0 (0)	7 (33)	

As independent validation of our observation, we identified patients with similar clinical data made available through a study of serous ovarian cancer by The Cancer Genome Atlas (TCGA) [32]. From data accessed on November 1, 2016, we found 214 of 603 patients with stage III HGSOC and completed overall survival data. Applying the same selection criteria used to filter the PM cohort, we identified 60 of 288 patients had primary platinum resistance and OS between 6 months and 2 years (28%), and 10 patients (5%) with extended platinum sensitivity and OS ≥ 10 years (Additional file 1: Figure S2B). Consistent with the PM cohort, the median age of diagnosis was lower for LT compared to ST patients (60.5 vs. 67 years median age at diagnosis). While CA125 levels were not available in the TCGA cohort clinical data, > 85% of ST survivors had measurable tumor burden post-surgery and 40% (4/10) LT patients had residual disease.

### High somatic mutation burden is associated with long-term survival in HGSOC

**Fig. 1:**
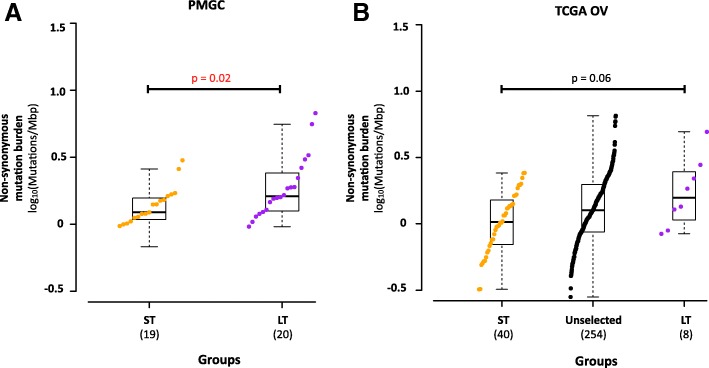
Somatic mutation burden of high-grade serous ovarian cancer exceptional short and long survivors. **a** Comparison of non-synonymous somatic mutation burden between exceptional short-term (*n* = 19) and long-term (*n* = 20) HGSOC survivor cohorts in this study. **b** Comparison of somatic mutation burden between exceptional short-term (*n* = 40) and long-term (*n* = 8) HGSOC survivor cohorts selected from the TCGA ovarian serous cystadenocarcinoma study. Non-synonymous mutation burden for each individual in each group is shown in increasing order. Data points are colored by group, short-term in orange, long-term in purple, and others in black. Boxplot for each group shows the group summary statistics for each survival group. Statistical significance is tested by non-parametric 2-sided Wilcoxon rank test for non-paired data and raw *p* value is reported. For TCGA, only difference between short- and long-term survivors is assessed

**Fig. 2:**
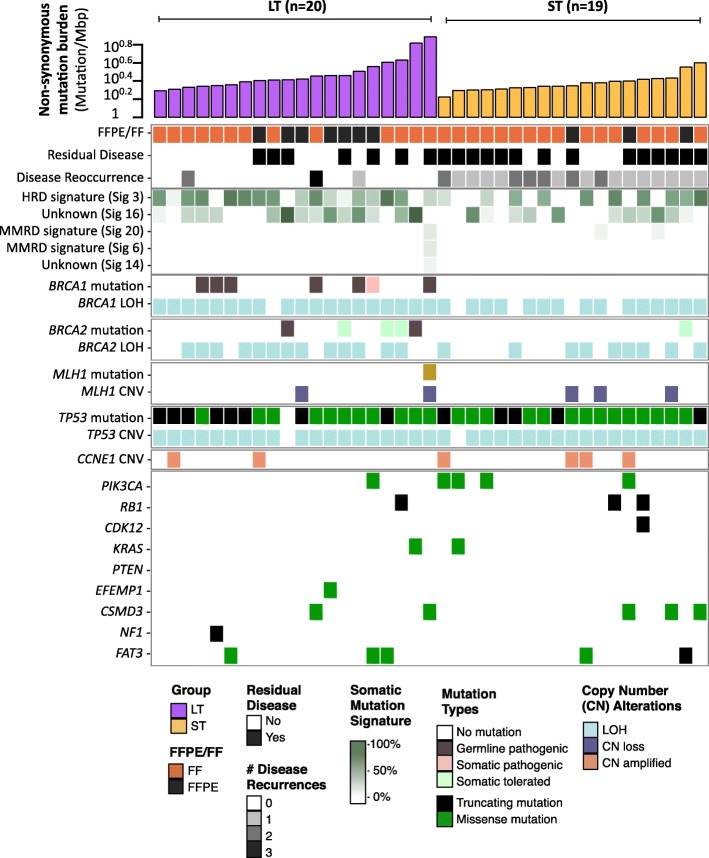
Landscape of genomic alterations in exceptional long- and short-term survivors of HGSOC. Summary of selected clinical and measured molecular characteristics by whole exome sequencing is shown for each primary tumor in the research cohort ordered by survival cohort and increasing somatic mutation burden. Mutations in genes found to be significantly recurrently mutated in HGSOC from the TCGA study are shown, with color for each alteration type illustrated in the legend

To identify genomic features associated with LT survival, we conducted exome and transcriptome analysis of 39 tumors at diagnosis and matched normal material from patients registered at PM (19 ST and 20 LT; 2 ST tumors from the clinical analysis were not included due to low-quality genomic data; Additional file 4: Tables S1, S2). Exomes were sequenced to median coverage 235× in tumors and 67× normal. Tumor transcriptomes were sequenced using a median 208 million reads. This analysis uncovered a median mutation frequency of 1.49 non-synonymous mutations per megabase (Fig. 1a) (range 0.678–6.740) consistent with TCGA report (Fig. 1b). In our cohort, and in the TCGA data, we found that mutation frequency was higher in LT versus ST samples (*p* = 0.022, median 1.62 vs. 1.22 non-synonymous mutations/Mbp). The tumor with the highest mutation burden was a carrier of a pathogenic *BRCA1* variant (p.Asn1236Phefs) and harbored two-hit somatic inactivation of *MLH1* through a truncating mutation (p.Ser170Argfs*20) coupled with loss of heterozygosity of chromosome 3p22.2 (Fig. 2), consistent with hypermutation seen in other cancers [33]. Increased mutation rate has been associated with enhanced immunogenicity in other tumors [34] and may explain increased survival in HGSOC. A long-term survivor patient in the TCGA cohort also carried a somatic *MLH1* mutation (p.Arg100Ter).

Consistent with genome landscape studies of HGSOC [32, 35, 36], *TP53* (38/39, 97%), *BRCA1* (7/39, 18%), and *BRCA2* (6/39, 15%) were the most frequently mutated genes in our cohort (Fig. 2). Genes mutated at lower frequencies in HGSOC (*CDK12*, *KRAS*, *PTEN*, *RB1*, *EFEMP1*, and *NF1*) were mutated in < 10% of our cohort, consistent with the TCGA data.

### Loss of BRCA1 or BRCA2 function is a molecular characteristic of long-term survival

We observed an enrichment of *BRCA1* and *BRCA2* mutations in the LT compared to the ST group (LT = 12/20, ST = 1/19, Fisher’s exact *p* = 0.0004) (Table 2). Pathogenic germline mutations in *BRCA1* and *BRCA2* are identified exclusively in the long-term survivors (*BRCA1* = 6, *BRCA2* = 2). Of the 5 somatic mutations identified in *BRCA1* and *BRCA2*, only 2 were truncation mutations that could result in loss of *BRCA1/2* function (*BRCA1* p.Trp1712Ter and *BRCA2* p.ThrAsp1867fs). All somatic mutations detected are also coupled with loss of heterozygosity (LOH) in the corresponding gene locus. One tumor from a ST patient had a somatic missense mutation in *BRCA2* (p.Pro2257Ser, MAF = 0.15) that is classified as tolerated and benign by SIFT (score = 0.12) and PolyPhen2 (score = 0.047), and therefore considered as non-pathogenic. This mutation has also never been reported in other tumors within the COSMIC database.

**Table 2:** Germline and somatic mutations in *BRCA1* and *BRCA2*

Patient ID	Group	Germline/somatic	Gene	Protein Change	MAF (normal)	MAF (tumor)	Pathogenic/Tolerated	LOH	COSMIC
LTS-004	LT	Germline	BRCA1	p.Q1111fs	0.42	0.75	Pathogenic	yes	
LTS-012	LT	Germline	BRCA1	p.V299fs	0.55	0.65	Pathogenic	yes	
LTS-017	LT	Germline	BRCA1	p.NIP1236fs	0.49	0.9	Pathogenic	yes	
LTS-019	LT	Germline	BRCA1	p.W1815*	0.45	0.85	Pathogenic	yes	
LTS-022	LT	Somatic	BRCA1	p.W1712*	0	0.5	Pathogenic	yes	
LTS-025	LT	Germline	BRCA1	p.S267fs	0.43	0.87	Pathogenic	yes	
LTS-029	LT	Germline	BRCA1	p.Q1756fs	0.46	0.91	Pathogenic	yes	
LTS-007	LT	Germline	BRCA2	p.V2527fs	0.32	0.43	Pathogenic	no	
LTS-013	LT	Somatic	BRCA2	p.TD1867fs	0	0.59	Pathogenic	yes	
LTS-021	LT	Somatic	BRCA2	p.N991D	0	0.74	Tolerated	yes	yes
LTS-023	LT	Somatic	BRCA2	p.S2835P	0	0.81	Tolerated	yes	yes
LTS-031	LT	Germline	BRCA2	p.D2242fs	0.65	0.68	Pathogenic	yes	
LTS-038	ST	Somatic	BRCA2	p.P2257S	0	0.15	Tolerated	no	no

**Fig. 3:**
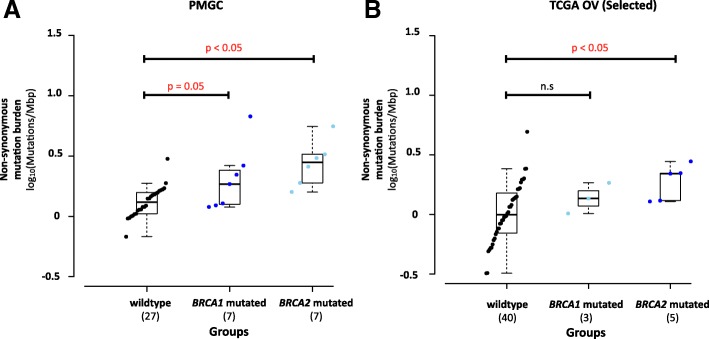
Mutation burden in *BRCA1*- and *BRCA2*-mutated HGSOC. **a** Comparison of somatic mutation burden between wild-type (no mutations detected, *n* = 27), *BRCA1* (*n* = 7), and *BRCA2* (n = 7)-mutated (germline and somatic) HGSOC in our study. **b** Comparison of somatic mutation burden between wild type (*n* = 40), *BRCA1* (*n* = 5) and *BRCA2* (*n* = 3) mutated (germline and somatic) in short- and long-term exceptional surviving HGSOC from the TCGA ovarian serous cystadenocarcinoma study. Mutation burden for each individual in each group is shown in increasing order. The patient with the highest mutation burden in the *BRCA1*-mutated group also has biallelic *MLH1* loss. Data points are colored by group, wild-type in black, *BRCA1*-mutated in dark-blue, and *BRCA2*-mutated in light-blue. Groups are sorted by increasing median mutation burden. Boxplot for each group shows the group summary statistics for each survival group. Statistical significance is tested by non-parametric 2-sided Wilcoxon rank test for non-paired data and raw *p* value is reported. n.s. *p* > 0.05

Overall, tumors with loss of function *BRCA1/2* mutations had a trend towards higher mutation frequency compared to tumors with intact *BRCA1/2* (*p* = 0.059) (Fig. 3a), with *BRCA2*-mutated tumors having the highest mutation burden, suggesting that defects in DNA homologous recombination repair may render the genome vulnerable to accumulating sequence mutations. We also observed a similar trend in the TCGA dataset (Fig. 3b).

While LOH in *BRCA1* was present in 88% (36/41) of all subjects (LT and ST) and frequently coupled with DNA copy loss (72%, 26/36), we did not observe significant loss or decrease of *BRCA1* gene expression in these samples as compared to samples without *BRCA1* copy loss (Additional file 1: Figure S3A). This observation could be confounded by wild-type *BRCA1* gene expression from contaminating normal tissue in the tumor specimen. Despite higher frequency of *BRCA1* loss of function mutations in the samples from LT cohort, no difference was seen in *BRCA1* transcript expression between the two survival groups. Similarly, *BRCA2* was most often affected by LOH (58%, 24/41 of all patients) and DNA copy loss across both survival groups (92%, 22/24) with no differences in gene expression between LT and ST groups (Additional file 1: Figure S3B).

### Spectrum and frequency of TP53 somatic mutations in LT and ST HGSOC

*TP53* mutations were prevalent across all HGSOC tumor samples (38/39, 97%, Table 3, Additional file 1: Figure S4A), and 39/41 tumors show loss of heterozygosity at the *TP53* locus. Through a combination of exome and deep-targeted sequencing, we detected 25 missense, 6 nonsense, 3 frame-shift deletion, 1 in-frame insertion, and 3 splice site mutations (Fig. 2 and Additional file 1: Figure S4A). A mutation in *TP53* was not detected in 1 LT patient, possibly due to a combination of low tumor cellularity (predicted 26% from Sequenza) and poor DNA quality from FFPE preservation. No differences in the frequencies of mutation types were observed between LT and ST. To assess the prognostic potential of *TP53* mutations, we categorized all mutations into 3 major categories as described by Brachova et al. [37]: 12/38 (32%) oncomorphic, 10/38 (26%) loss of function (LOF), and 16/38 (42%) unclassified *TP53* mutations. There was no statistical significant difference in the frequency of oncomorphic mutations between LT and ST cohorts (ST: 6/19, LT: 6/20, *p* = 0.72), although both cohorts harbored a significant fraction of unclassified mutations (ST: 9/19, LT: 7/20) (Additional file 1: Figure S4C). Therefore, further characterization of *TP53* mutations in LT and ST cohorts is needed to establish the function of these mutations.

**Table 3:** *TP53* Mutations in Study Cohort

Patient ID	Group	Variant type	Mutation protein change	Mutant allele fraction	Function affected	Oncomorphic?	Detection method
LTS-001	LT	Nonsense	p.S183*	0.47		no	Mutect
LTS-002	ST	Missense	p.E224D	0.27		no	Mutect
LTS-003	ST	Missense	p.R175H	0.83	Structural Change	yes	Mutect
LTS-004	LT	Frame Shift Del	p.P223fs	0.45		no	Strelka
LTS-005	ST	Missense	p.D281E	0.75		no	Mutect
LTS-006	ST	Missense	p.Y220C	0.46	Structural Change	yes	Mutect
LTS-007	LT	Missense	p.I195T	0.15		no	Strelka SNV/None by targeted seq
LTS-008	ST	Missense	p.C242F	0.64		no	Mutect
LTS-009	ST	Missense	p.M237I	0.57		no	Mutect
LTS-010	ST	Missense	p.Y220C	0.89	Structural Change	yes	Mutect
LTS-011	LT	Missense	p.R248Q	0.51	Structural Change	yes	Mutect
LTS-012	LT	Missense	p.R248Q	0.76	Structural Change	yes	Mutect
LTS-013	LT	Frame Shift Del	p.A70fs	0.57		no	Varscan2/Targeted Sequencing
LTS-014	LT	Splice Site	c.e7+1	0.89		no	Strelka SNV/Targeted Sequencing (g.chr17:7577498C > A)
LTS-015	ST	Splice Site	c.e8+1	0.74		no	Mutect/Strelka SNV
LTS-016	LT	Missense	p.R248Q	0.82	Structural Change	yes	Mutect
LTS-017	LT	Missense	p.I195T	0.7		no	Mutect
LTS-018	ST	Missense	p.G266E	0.73		no	Mutect
LTS-019	LT	Missense/Frame shift Ins	p.K139Q/ p.V143fs	0.72		no	Mutect/Strelka
LTS-020	LT	Splice Site	p.Q331Q	0.62		no	Mutect
LTS-021	LT	Missense	p.R248W	0.39	DNA binding	yes	Mutect
LTS-022	LT	Missense	p.G245S	0.72	Structural Change	no	Mutect
LTS-023	LT	Missense	p.T125P	1		no	Exome & Targeted sequencing
LTS-024	ST	Missense	p.R282W	0.6	Structural Change	no	Mutect
LTS-025	LT	Missense	p.R273H	0.91	DNA binding	yes	Targeted Sequencing
LTS-026	ST	Nonsense	p.E349*	0.46		no	Mutect
LTS-027	LT	Nonsense	p.R196*	0.56		no	Mutect
LTS-028	ST	Nonsense	p.G266*	0.93		no	Mutect
LTS-029	LT	Missense	p.Y163H	0.73		no	Mutect
LTS-030	LT	Missense	p.R273C	0.67	DNA binding	yes	Mutect
LTS-031	LT	Not detected	Not detected	–		no	None detected by WES on all callers/poor RNAseq
LTS-032	LT	Nonsense	p.W146*	0.86		no	Mutect
LTS-033	ST	Missense	p.R175H	0.4	Structural Change	yes	Mutect
LTS-034	ST	Missense	p.R273L	0.8		yes	Also found in normal (transformed adjacent normal)
LTS-035	ST	In Frame Insertion	p.266_267insLG	0.18	DNA binding	no	Strelka Exome & RNAseq
LTS-037	ST	Frame Shift Del	p.P87fs	0.77		no	Strelka
LTS-038	ST	Missense	p.R175H	0.63	Structural Change	yes	Mutect
LTS-039	ST	Missense	p.F270S	0.68		no	Strelka SNV
LTS-040	ST	Nonsense	p.E204*	0.51		no	Mutect

Consistent with known mutation spectra in *TP53*, 30 of 38 mutations were located within the p53 DNA-binding domain with oncomorphic p.Arg248 having the highest mutation frequency (4/29, 3 Arg > Gln, 1 Arg > Trp) (Additional file 1: Figure S3A). While p.Arg248 mutations occurred exclusively in tumors from LT survivors in our cohort, these mutations occurred exclusively in 4 ST patients in the TCGA cohort (Additional file 1: Figure S3B). Between the three categories of *TP53* mutations, we observed that tumors containing oncomorphic *TP53* mutations have the highest *TP53* mRNA expression (two-sided Wilcoxon Rank Sum: oncomorphic vs LOF (median expression log2(TPM + 1): 4.34 vs. 2.18, *p* = 0.008); oncomorphic vs unclassified (median expression log2(TPM + 1): 4.34 vs. 3.73, *p* = 0.22) (Additional file 1: Figure S4D). We observed a broad range of *TP53* mRNA expression in tumors with unclassified mutations. This observation further suggests that the unclassified set of *TP53* missense mutations may contain additional oncomorphic mutations that may come to light with further functional characterization of these variants.

### Short-term survivors lack BRCAness

Alexandrov et al. [38] described 20 distinct mutational signatures based on the frequency of somatic base substitution events and the flanking sequence context. To better understand the underlying mutational processes in our cohort, we determined the composition of mutational signatures by applying non-negative matrix factorization from the catalog of somatic mutations identified in each tumor. Signature 3 (BRCA signature), associated with inactivating *BRCA1* or *BRCA2* mutations in breast and pancreatic cancers and prevalent in ovarian cancer [35], is present in 27/39 samples. However, not all LT tumors are positive for signature 3. This observation suggests that presence of a BRCA-associated signature alone is not prognostic in HGSOC (Fig. 2). The BRCA signature occurs less frequently in short-term survivors (ST vs LT, 10/19 vs 17/20, fisher’s exact test *p* = 0.04), suggesting that lack of BRCAness [39] may be associated with poor survival in HGSOC (Additional file 1: Figure S6). Signature 16, possibly associated with active DNA repair by transcription-coupled nucleotide excision repair, is the dominant signature in tumors that have germline *BRCA2* mutations. Mutation signature associated with DNA mismatch repair deficiency and high mutation frequency (Signatures 20, 6, and 14) was only evident in the high mutation burden tumor with both *BRCA1* and *MLH*1 inactivation.

### HRD-LOH in short- and long-term survivors

All tumors exhibit highly altered karyotype with evidence of genome doubling (average estimated ploidy of 2.5 and 2.8, respectively for long- and short-survival) with frequent chromosome alterations characteristic of HGSOC including arm-level gains in 1p, 3q, 6p, and 20q, and losses in 4p, 4q, 6q 8p, 8q, 9q, 11p, 11q, 13q, 16p, 16q, 17p, 17q, 18q, 19q, 21q, and 22q (Additional file 1: Figures S7 and S8). All of the frequently detected arm-level events in our cohort were previously reported by the TCGA. Two hundred fifteen and 156 unique genes within focal amplification regions were found in long- and short-term samples, respectively using GISTIC2.0 algorithm [23] (Additional file 1: Figure S9). One of these genes, *CCNE1*, is focally amplified in 4/19 ST and 2/20 LT survivor tumors. The increased frequency of *CCNE1* gain in patients with short survival time is consistent with its known association with poor prognosis in ovarian cancer [40]. However, *CCNE1* amplification has also been observed in long-term survivors within the TCGA cohort at a 10% (1/10) frequency.

We also compared frequencies of copy number alterations in 5 genomic regions (19q12 amplification, 14q32.33 amplification, 3q29 amplification, 20q13.21-q13.32 amplification, and 20q13.2 amplification) previously associated with ovarian cancer survival [41–43]. In this analysis, only amplification of 19q12 (containing *CCNE1*) was frequently altered in ST and not in LT.

**Fig. 4:**
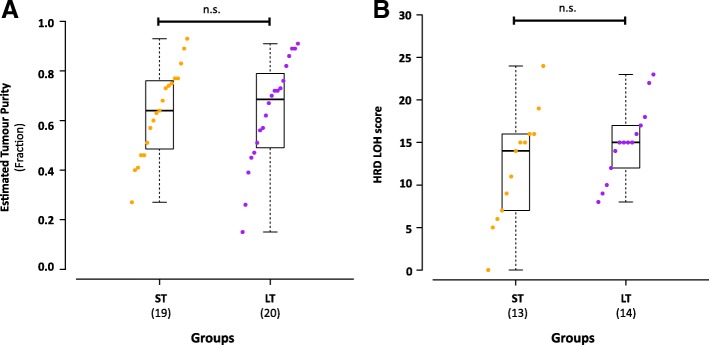
Homologous recombination deficiency in exceptional short- and long-term HGSOC survivors. **a** Comparison of estimated tumor cell cellularity in the sequenced tumor tissue between long- (*n* = 20) and short- (*n* = 19) term HGSOC in this study. **b** Comparison of whole exome sequencing data derived HRD-LOH scores from tumors with greater than 50% tumor cellularity between exceptional survivor groups (long-term = 14, short-term 13). Individual data points in each group is shown in increasing order. Data points are colored by group, short-term in orange and long-term in purple. Boxplot for each group shows the group summary statistics for each survival group. Statistical significance is tested by non-parametric 2-sided Wilcoxon rank test for non-paired data and raw *p* values are reported. n.s. *p* > 0.05

To evaluate reported prognostic value of DNA homologous repair deficiency in HGSOC [44, 45], we compared homologous recombination deficiency-loss of heterozygosity (HRD-LOH) score between LT and ST tumors. While we did not observe significant difference between the estimated tumor cellularity of LT and ST groups (Fig. 4a), we have observed lower sensitivity of CNA detection in tumors with low cellularity. To mitigate the effects of tumor cellularity, we only selected tumors with > 50% (LT *n* = 14, ST *n* = 13) cellularity for the HRD-LOH comparison. While more ST tumors have lower HRD-LOH score, no significant difference is observed between LT and ST groups (Fig. 4b). A larger range of HRD-LOH score is seen in the ST group (0–24) as compared to LT (8–23). This suggests the existence of other uncharacterized mechanisms that contribute to genomic instability and survival in HGSOC beyond *BRCA1/2* disruption.

### Increased tumor immune-reactivity and immune cell infiltration are features of LT HGSOC

**Fig. 5:**
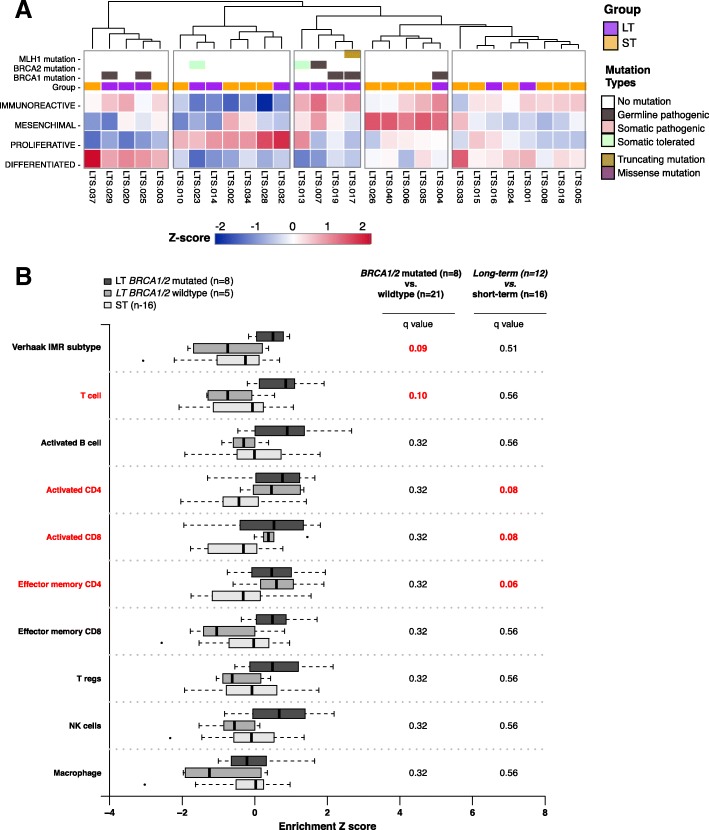
Inference of tumor microenvironment in exceptional short- and long-term survivors of HGSOC. **a** Heat-map of TCGA/Verhaak HGSOC gene-expression subtype scores for 29 fresh-frozen preserved primary tumor tissues in our study group (long-term survival = 13, short-term survival = 16). The display order of tumors is determined by unsupervised hierarchical clustering the *z*-score normalized HGSOC gene-expression subtype score profiles. Mutations in DNA damage repair genes (*BRCA1, BRCA2*, and *MLH1*) and survival groups are annotated in color tracks above the heatmap. Annotation colors are shown in the legend. **b** Comparison of enrichment of cellular components within the tumor immune microenvironment between long-term and short-term survivors with or without mutations in *BRCA1* and *BRCA2*. Enrichment of selected immune cellular components is inferred from available RNA-seq gene-expression profiles and publicly available cell-type-specific gene sets by ssGSEA. Boxplots for each group, long-term with *BRCA1/2* mutation (*n* = 8, dark-grey), long-term without *BRCA1/2* mutation (*n* = 5, medium-grey), and short-term without *BRCA1/2* mutation (*n* = 16, light-grey), show the summary statics. Statistical significance is tested by non-parametric 2-sided Wilcoxon rank test for non-paired data between long-term surviving *BRCA1/2* mutated group (*n* = 8) to all *BRCA1/2* not-mutated group (*n* = 21), and between long- (*n* = 13) to short- (*n* = 16) term survivors. *p* values are multiple-testing corrected (false discovery rate) and *q* values are presented. *q* values ≤ 0.1 are high-lighted in red

To assess relationships of immune cell infiltration with survival, we assessed enrichment of four published gene expression subtypes (including an immuneoreactive subtype, IMR) [26] as well as a total immune cell infiltration score (ESTIMATE algorithm) [27] in 29 tumors with available RNA-seq data (13 LT and 16 ST). Consistent with previous reports, all tumors showed enrichment in more than one gene expression subtype (Fig. 5a). Through unsupervised hierarchical clustering of each tumor by the gene-expression subtype score profiles, it was evident that a group of 4 *BRCA1/2* mutated tumors, characterized by high immunoreactive subtype score, formed a unique cluster. We also observed a cluster of tumors characterized by strong mesenchymal expression subtype signature containing almost exclusively of short-term ST survivors (*n* = 4/5) with the exception of one long-term survivor that also exhibited strong immunoreactive signature. The remaining 4 clusters contain various proportion of LT and ST members, illustrating the complexity of the underlying molecular pathology of HGSOC.

While we did not observe a statistically significant difference in immune scores between LT and ST tumors across the cohort (two-sided Wilcoxon Rank Sum, *n* = 13 vs 16, mean = 1.6 vs 1.5, *p* = 0.170) (Fig. 5b), more LTs than STs were amongst the top 25% of tumors with the highest ESTIMATE Immune score (fisher’s exact test *p* = 0.027). Focusing on *BRCA1/2*-mutated tumors, we found higher immune enrichment scores compared to tumors with wild-type *BRCA1*/2 (two-sided Wilcoxon Rank Sum, *n* = 7 vs 22, mean = 1.7 vs 1.5, *q* = 0.09) (Fig. 5).

As specific immune cell types in the tumor microenvironment may underlie LT survival, we also assessed the role of 8 immune cell populations previously associated with survival outcome in various cancer types, including HGSOC [12, 13, 28, 46]. Using ssGSEA [25], we found LT tumors were enriched for activated CD8^+^ T (*q* = 0.08), activated CD4^+^ T (*q* = 0.08), and effector memory CD4^+^ T cells (*q* = 0.06) (Fig. 5b). To further illustrate the independence of cell-type specific infiltration from total immune enrichment, we found enrichment scores of activated CD8^+^ T cells, activated CD4^+^ T cells, and effector memory CD4^+^ T cells were not correlated with total immune or immune reactivity scores (Pearson correlation < 0.5, *p* > 0.05, Additional file 1: Figure S10C, D, E). LT and ST showed no difference in enrichment of effector memory CD8^+^, regulatory T cells, activated B cells, macrophages, and NK cells (Fig. 5b), although this may be due to a lack of adequate reference gene sets or low frequency in the tumor microenvironment for these cell types.

From the TCGA ovarian cancer cohort, we identified 8 LT and 32 ST tumors that matched the survival selection criteria of our cohort. Here, we observed a similar trend of increased activated CD8^+^ T, CD4^+^ T, and effector memory CD4^+^ T cell gene-set enrichment between LT and ST tumors. This observation provided additional support to suggest that increased activated CD8^+^ and CD4^+^T lymphocytes in the tumor microenvironment may play an important role in improved LT survival outcome in HGSOC (Additional file 1: Figure S11). We also confirmed no difference in enrichment of macrophages, effector memory CD8^+^ T cells, NK cells, or regulatory T cells between LT versus ST TCGA tumors (Additional file 1: Figure S11).

### *ESR1-CCDC170* is a novel recurrent gene fusion in HGSOC with short survival

**Fig. 6:**
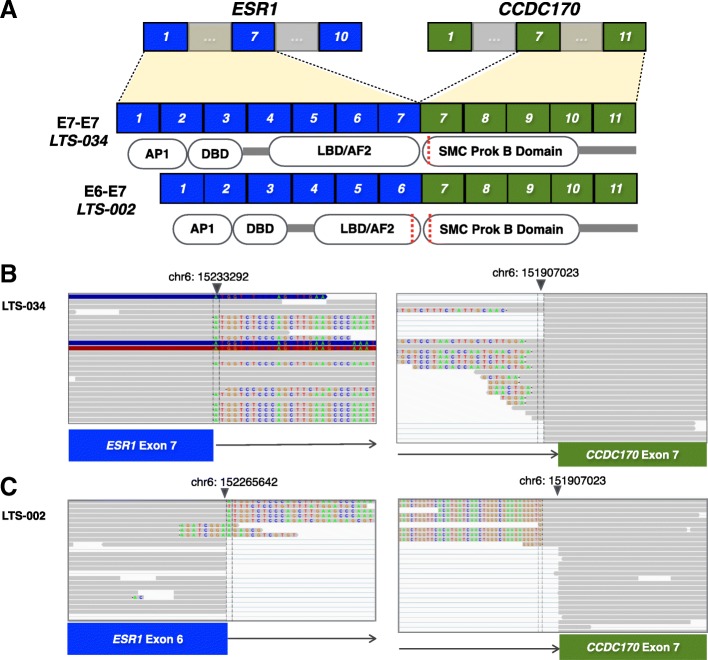
Recurrent *ESR1*-*CCDC170* gene fusion in exceptional short-term surviving HGSOC. **a** Schematic diagram of the exons from *ESR1* and *CCDC170* included within the detected gene-fusion mRNA by RNA-seq in the two HGSOC primary tumor tissues from exceptionally short-term surviving patients. Diagram of protein domains encoded by the retained exons is shown for each fusion. **b** RNA-seq reads supporting the *ESR1*-*CCDC170* fusion mRNA in patient LTS-034. **c** RNA-seq reads supporting the *ESR1*-*CCDC170* fusion mRNA in patient LTS-002. Portions of the junction-spanning reads that align to the reference sequence of *ESR1* and *CCDC170* are colored in grey and the mismatched bases are shown in color

Fusion gene RNA transcripts were predicted for 13 LT and 16 ST HGSOC from the RNAseq data. Of the 125 total potential fusions involving different gene partner pairs identified, 4 candidate fusions (*ESR1-CCDC170*, *DLEU1-DLEU7, KMT2E-LHFPL3*, and *LOC101928103-ABAC12*) were recurrent (occurred in two or more tumors) (Additional file 4: Table S3). *ESR1-CCDC170*, present in 2 ST patients, while has never been reported in HGSOC, is the most frequent gene-fusion (6–8%) found in luminal B breast cancer with poor clinical prognosis [47] (Fig. 6). *DLEU1-DLEU7*, present in 2 LT and 1 ST patient, has not been previously reported in HGSOC or other cancer types (Additional file 1: Figures S12-S14). However, increased *DLEU1* expression has been shown to sequester the tumor suppressor function of miR-290-3p and increase growth and invasiveness of ovarian cancer cell lines in vitro [48]. This fusion product lacks the predicted miR-290-3p binding sequence and therefore may provide a new mechanism to control HGSOC aggressiveness in vivo.

## Discussion

With limited number of approved treatments for managing HGSOC, long-term survival is strongly dependent on the extent and duration of chemosensitivity in the cancer cells. Beyond *BRCA1/2* mutation status, no other biomarker enables up-front and precise identification of patients with platinum sensitive or resistant disease. As such, initial treatment plans are not informed by the underlying disease biology. Given the high rate of relapse following initial treatment in HGSOC, several trials are on-going to add anti-angiogenics, PARP and/or PDL-1 inhibitors to standard chemotherapy in the hope to increase the progression free and overall survivals. However, identification of mechanisms of inherent platinum resistance and platinum sensitivity will enable the discovery of biomarkers that may be further validated in this new trials approach. By comparing molecular characteristics of primary advanced HGSOC from patients who experienced prolonged chemosensitivity (OS > 10 years) to patients with primary chemoresistance (OS < 2 years), we sought to uncover factors that may be used for treatment decision in HGSOC. Currently, the strongest predictors of LT survival remain the disease stage and no residual disease post-surgery [49]. Consistent with this finding, the majority of our LT patients had complete disease resection (Table 1, Fig. 2). While initial tumor burden measured by CA125 serum levels did not predict exceptional survival, low serum CA125 levels post-treatment (surgery and chemotherapy) are associated with long-term survival. Specifically, CA125 levels for all long-term responders fell to less than 10 units/mL post-chemotherapy, suggesting that these tumors are highly sensitive to standard of care treatment. This finding provides additional evidence that CA125 kinetics may have predictive value and may be used as a tool in drug response assessment [50, 51].

Previous studies in HGSOC have focused on describing mutational processes that contribute to tumorigenesis, molecular signatures that correlate to survival and mechanisms of chemoresistance. However, most of these studies rely on limited survival data with less than 5 years of patient follow-up. Our cohort with greater than 10 years of follow-up confirms that biallelic inactivation of *BRCA1* or *BRCA2*, through either germline or somatic mutation, coupled with loss of heterozygosity, is associated with extended long survival (Fig. 2, Table 2). The association of *BRCA1/2* mutations with improved OS and progression-free survival has been previously reported in ovarian cancers [9]. Biallelic inactivation of *BRCA1* was reported as a potential mechanism of long-term response to Olaparib, a PARP inhibitor, in a HGSOC patient with > 7 years response [51]. Interestingly, the only *BRCA2* somatic mutation detected in the short-term survivor patient had low mutant allele frequency (MAF = 0.15) and retained the wildtype allele. The intact wildtype *BRCA2* allele may provide material for somatic *BRCA*1/2 recovery by copy number gain or upregulation to facilitate chemotherapy resistance and disease progression. Additionally, while there exists an enrichment of *BRCA1/2* abnormalities in the LT patients, not all LT patients harbor *BRCA1/2* mutations, suggesting alternate mechanisms conferring prolonged chemosensitivity are present in these tumors [6].

BRCAness is a term coined to describe tumors exhibiting phenotypes that are similar to those with loss of *BRCA1/2* function in the absence of a *BRCA1/2* mutation [39]. With the success of PARP inhibitors for patients with *BRCA1/2* mutation-positive ovarian cancers [7, 8, 52], the focus is now on identifying other molecular abnormalities that may confer “BRCAness” to tumors without apparent *BRCA* mutations. We hypothesize that LT tumors, regardless of *BRCA* mutation status, exhibit more characteristics of homologous repair deficiency as compared to the ST patients. We measured features of BRCAness by overall mutation burden, identifying mutations in other genes involved in DNA homologous recombination repair, inferring *BRCA* mutational signature and the homologous recombination deficiency loss of heterozygosity (HRD-LOH) score for each tumor from exome profiles [44, 45]. We identified higher number of non-synonymous mutations in LT compared to ST, consistent with higher mutation burden in *BRCA1/2* deficient tumors. Unlike previous reports, we did not identify an enrichment of loss of function mutations in other HR genes in our study cohort [35], probably given the small size of our study cohort and the low frequency of non-*BRCA* HR gene mutations in HGSOC. However, a mutational signature associated with BRCA inactivation is prevalent in both LT and ST groups (total 28/39 tumors). Although both survival groups have high percentage of *BRCA* mutation signatures, the tumors from short-term survivors are enriched within the tumors lacking this signature. In addition, tumors with low HRD-LOH scores are enriched with ST patients. Together, findings suggest absence of BRCAness may be a prognostic characteristic of poor survival in HGSOC.

Given the prevalence of *TP53* mutations in HGSOC, it was suggested that some non-synonymous mutations may provide survival advantage to tumor cells and associated with poor patient survival [37]. By over-expressing specific *TP53* mutations in *TP53−/−* ovarian cancer cell lines in vitro or by measuring tumorigenesis in mouse and rat models, studies have demonstrated a subset of mutations that increase chemo-resistance and promote cancer cell growth [37]. Our analysis of this subset of oncomorphic mutations did not uncover enrichment in LT versus ST tumors. However, both cohorts contained a substantial number of unclassified variants expressed at differing levels, suggesting further characterization of these mutations is warranted.

Increased lymphocytic infiltration in the tumor microenvironment is a histological phenotype observed in *BRCA1/2*-mutated ovarian tumor [53]. The association of infiltrating immune cells and patient survival is strongly dependent on quantity and the composition of cell types present [10, 12]. As such, B cells, CD4^+^, and CD8^+^ T cells have been associated with improved clinical outcomes whereas regulatory cell types, such as regulatory T cells and neutrophils, have been associated with poor outcome in ovarian, breast, lung, and colon cancers [54–57]. Therapeutic strategies to increase the quantities of infiltrating immune cells with tumor-killing abilities such as immune-checkpoint inhibition and adoptive cell transfer therapies have been at the forefront of clinical trials and research in recent years. Using whole transcriptome analysis and publically available gene sets, we inferred the enrichment of lymphocytic infiltration as a whole, as well as of individual subtypes of immune cells for each tumor specimen. Using this method, we confirmed that the immune-reactive subtype of HGSOC is correlated with the immune score measure from ESTIMATE and both are higher in LT tumors. We also observed an increase in immune score in *BRCA1/2*-mutated tumors compared to *BRCA1/2* wild-type tumors. This trend is consistent when comparing LT to ST groups, in which activated CD4^+^, CD8^+^, and effector CD4^+^ T lymphocytes were enriched in LT tumors; however, these gene set scores did not correlate directly with bulk immune scores. This observation suggests that the presence of specific cells in the microenvironment may contribute directly to eliminating tumor cells or increasing chemosensitivity, with or without the involvement of *BRCA* inactivation by mutation. In addition, we observed a small group of ST tumors with high mesenchymal gene-expression subtype scores. The mesenchymal subtype was described by Tothill et al. who showed that HGSOCs within this molecular subgroup had poorer overall survival as compared with those defined by other molecular subtypes [58]. A recent study showed that HGSOC tumors with mesenchymal gene-expression subtype are associated with disseminated intraperitoneal disease and lower rates of complete tumor resection [59]. Together, these studies further suggest that mesenchymal HGSOCs have poor clinical outcomes. A recent retrospective analysis showed that mesenchymal HGSOC tumors may respond favorably to anti-angiogenic treatment, providing an option for targeted therapy in this specific subgroup [60].

While clinical and molecular factors contributing to chemo-resistance in HGSOC have been described, recurrent gene-fusions in HGSOC associated with therapeutic outcome have yet to be replicated across multiple studies [35]. Using RNA-seq in our small study cohort, we identified the *ESR1-CCDC170* fusion, previously reported in aggressive luminal B breast cancers, in 2/16 short-term survivors. In vitro experiments showed increases in cellular proliferation and migration when *ESR1-CCDC170* fusions are expressed in the MCF10A breast epithelial cell-line. The presence of this variant within exceptionally short-term survivors with platinum resistance may point to a novel mechanism that contributes the aggressive oncogenic phenotype in these tumors. Further functional validations will have to be performed in other HGSOC cohorts in future investigations.

## Conclusions

In this comprehensive analysis, we focused on comparing treatment-naïve primary HGSOC tumor from two groups of patients selected based on their extreme differences in OS. We have demonstrated that compared to primary chemoresistant HGSOC, LT survival in HGSOC can be characterized by elevated mutation burden, biallelic inactivation of *BRCA1* or *BRCA2,* and increased CD4^+^ and CD8^+^ lymphocytic infiltration in the tumor microenvironment. We are also the first to report the *ESR1-CCDC170* gene fusion in tumors from two HGSOC patients with extremely short survival. Identifying mechanisms involved in the response or resistance to treatment is essential to devising precision treatment plans, and future strategies will likely rely on multiple clinical and immunogenomic factors. With only a small group of patients, this study is exploratory and hypothesis generating in nature and will require validation by future studies. However, this analysis of exceptional responders in HGSOC has the potential to contribute to our understanding of the biology of ovarian cancer, with the goal of improving the survival of patients [61, 62]. Given the molecular heterogeneity that exists within HGSOC, we suggest that optimal patient care should be provided through a multidisciplinary longitudinal approach that integrates expertise from meaningful tumor characterizations such as *BRCA1/2* mutation status, mutation burden, HR deficiency, and tumor microenvironment immune composition at the time of diagnosis and relapse [7, 8, 63].

## Additional files

Additional file 1: Supplementary figures for the manuscript. (PDF 14062 kb)

Additional file 2: Supplementary methods for the manuscript. (PDF 112 kb)

Additional file 3: CNV segment size as distribution per sample. Distribution of CNV segment size as percentage of chromosome arm in each sequenced tumor sample. Sequenza estimated sample ploidy and the threshold used for determining copy number amplification is shown for each sample as colored horizontal lines. (PDF 34 kb)

Additional file 4: Supplementary tables for the manuscript. (XLSX 60 kb)

Additional file 5: All Strelka indels. All Indels called by Strelka and annotated by Oncotator. (TXT 3458 kb)

Additional file 6: All CNV segs annotated. All CNV segs generated by Sequenza and annotated by Oncotator. (TXT 15904 kb)

Additional file 7 All Mutect mutations 1. SNV mutations called by Mutect and annotated by Oncotator for samples LTS-001_T, LTS-002_T, LTS-003_T, LTS-004_T, LTS-005_T, LTS-006_T. (TXT 16479 kb)

Additional file 8: All Mutect mutations 2. All SNV mutations called by Mutect and annotated by Oncotator for samples LTS-007_T in chromosomes 1 to 10. (TXT 17783 kb)

Additional file 9: All Mutect mutations 3. All SNV mutations called by Mutect and annotated by Oncotator for samples LTS-007_T in chromosomes 11 to 22, M, X, and Y. (TXT 12679 kb)

Additional file 10: All Mutect mutations 4. All SNV mutations called by Mutect and annotated by Oncotator for samples LTS-008_T, LTS-009_T, LTS-010_T, LTS-011_T, LTS-012_T, LTS-013_T, LTS-014_T, LTS-015_T, LTS-016_T. (TXT 13456 kb)

Additional file 11: All Mutect mutations 5. All SNV mutations called by Mutect and annotated by Oncotator for samples LTS-017_T, LTS-018_T, LTS-021_T. (TXT 18408 kb)

Additional file 12 All Mutect mutations 6. All SNV mutations called by Mutect and annotated by Oncotator for samples LTS-019_T, LTS-020_T, LTS-022_T, LTS-023_T, LTS-024_T, LTS-025_T, LTS-026_T, LTS-027_T, LTS-028_T. (TXT 19235 kb)

Additional file 13: All Mutect mutations 7. All SNV mutations called by Mutect and annotated by Oncotator for samples LTS-029_T, LTS-030_T, LTS-031_T. (TXT 14352 kb)

Additional file 14: All Mutect mutations 8. All SNV mutations called by Mutect and annotated by Oncotator for samples LTS-032_T, LTS-033_T, LTS-034_T, LTS-035_T, LTS-037_T, LTS-038_T, LTS-039_T, LTS-040_T. (TXT 8415 kb)

## Abbreviations

ABAC12: ATP-binding cassette, subfamily A, member 12
BRCA1: Breast Cancer Gene 1
BRCA2: Breast Cancer Gene 2
CA125: Cancer Antigen 125
CCDC170: Coiled-coil domain containing 170
CCNE1: Cyclin E 1
CD4: Cluster of differentiation 4
CD8: Cluster of differentiation 8
CDK12: Cyclin dependent kinase 12
CNV: Copy number variation
DLEU1: Deleted in lymphocytic leukemia 1
DLEU7: Deleted in lymphocytic leukemia 7
DNA: Deoxyribose nucleic acid
EFEMP1: EGF containing fibulin-like extraceullular matrix protein 1
EOC: Epithelial ovarian cancer
ESR1: Estogen receptor 1
FDR: False discovery rate
FF: Fresh frozen
FFPE: Formalin-fixed paraffin embedded
FIGO: International Federation of Gynecology and Obstetrics
GATK: Genome analysis toolkit
HGSOC: High-grade serous ovarian cancer
HR: Homologous recombination
HRD: Homologous recombination deficiency
IMR: Immuneoreactive
KMT2E: Lysine methyltransferase 2E
KRAS: Kristen rat sarcoma viral oncogene homolog
LHFPL3: Lipoma HMGIC fusion partner-like 4
LOH: Loss of heterozygosity
LT: Long term
MAF: Mutant allele frequency
Mbp: Million base pairs
miRNA: MicroRNA
MLH1: MutL homolog 1
NF1: Neurofibromin 1
NK: Natural killer
OS: Overall survival
PARP: Poly-ADP polimerase
PM: Princess Margaret
PTEN: Phosphatase and tensin homolog
RB1: Retinoblastoma 1
RNA: Ribose nucleic acid
SCNA: Somatic copy number alteration
SNV: Single nucleotide variant
ssGSEA: Single sample geneset enrichment analysis
ST: Short term
TCGA: The Cancer Genome Atlas
TP53: Tumor protein 53
TPM: Transcripts per million
Tregs: Regulatory T cells
VAF: Variant allele frequency
WES: Whole exome sequencing
