# Supplementary material for: Landscape of genomic alterations in high-grade serous ovarian cancer from exceptional long- and short-term survivors
Source: Genome Med. 2018 Oct 31;10:81. doi: 10.1186/s13073-018-0590-x (PMC6208125; doi:10.1186/s13073-018-0590-x)
Supplement: Supplementary file 1 — Supplementary figures for the manuscript. (PDF 14062 kb) [file 13073_2018_590_MOESM1_ESM.pdf]

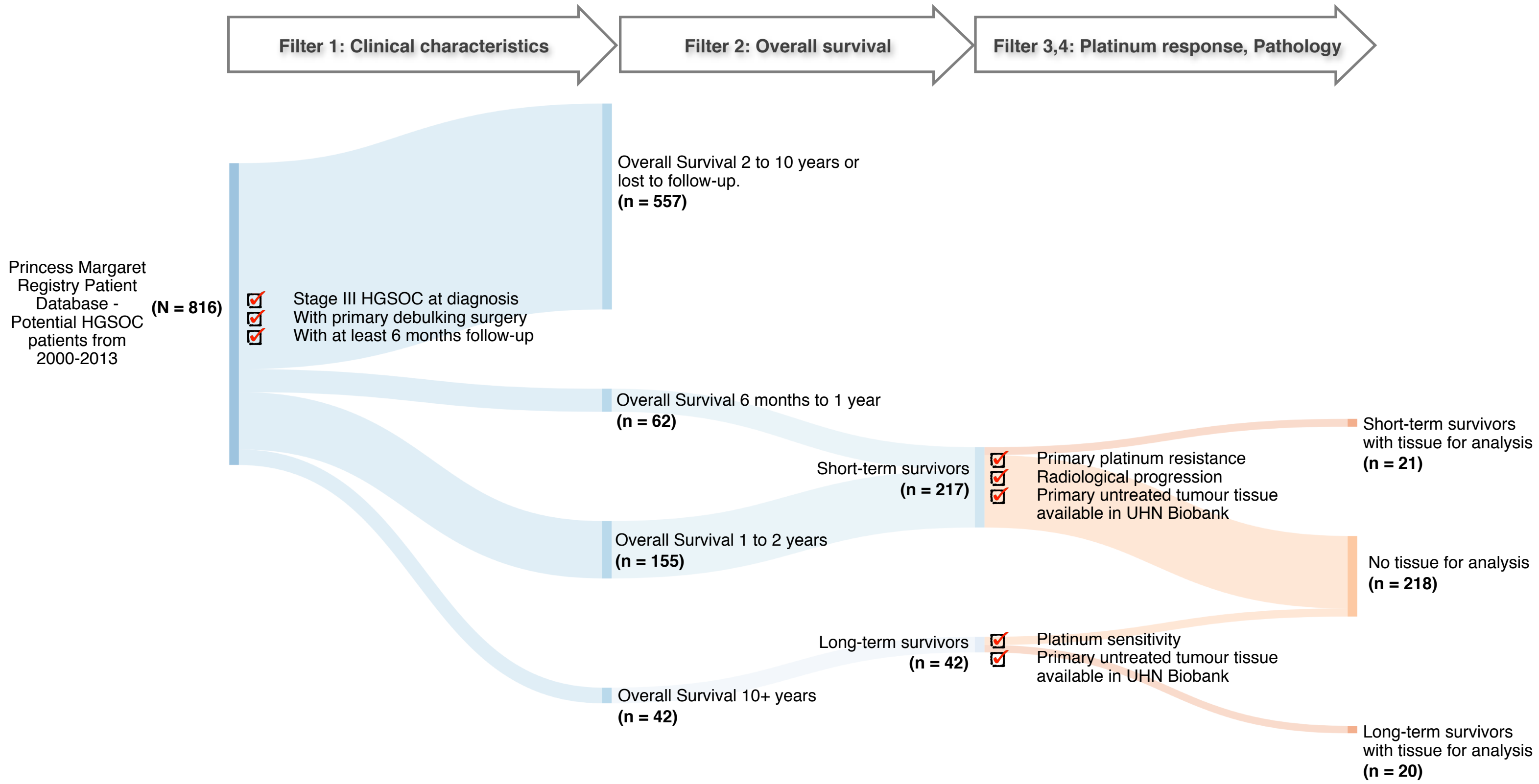

Figure S1. Patient and tissue selection flow for the study.

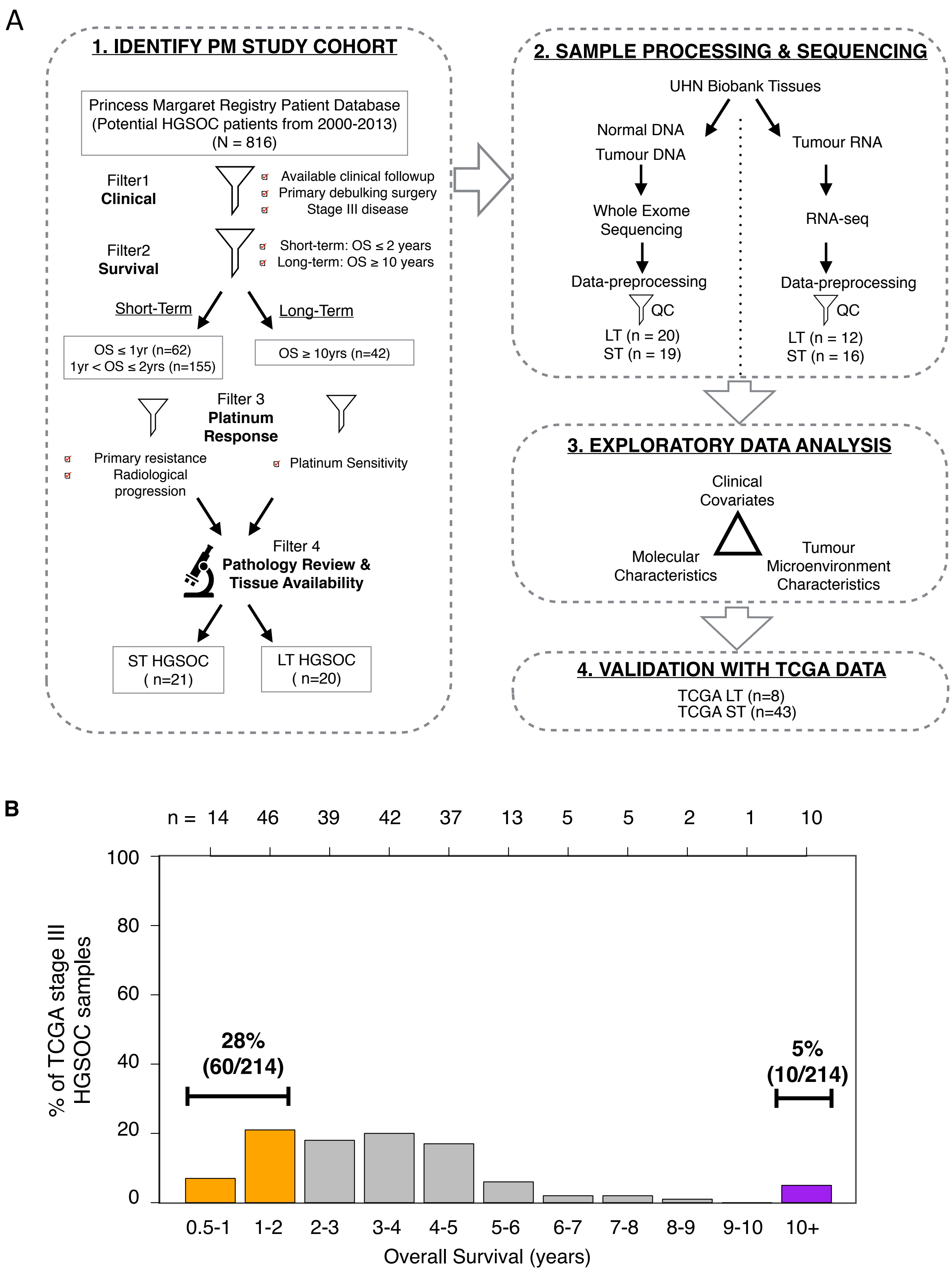

Figure S2. Flowchart of study design and analysis workflow.

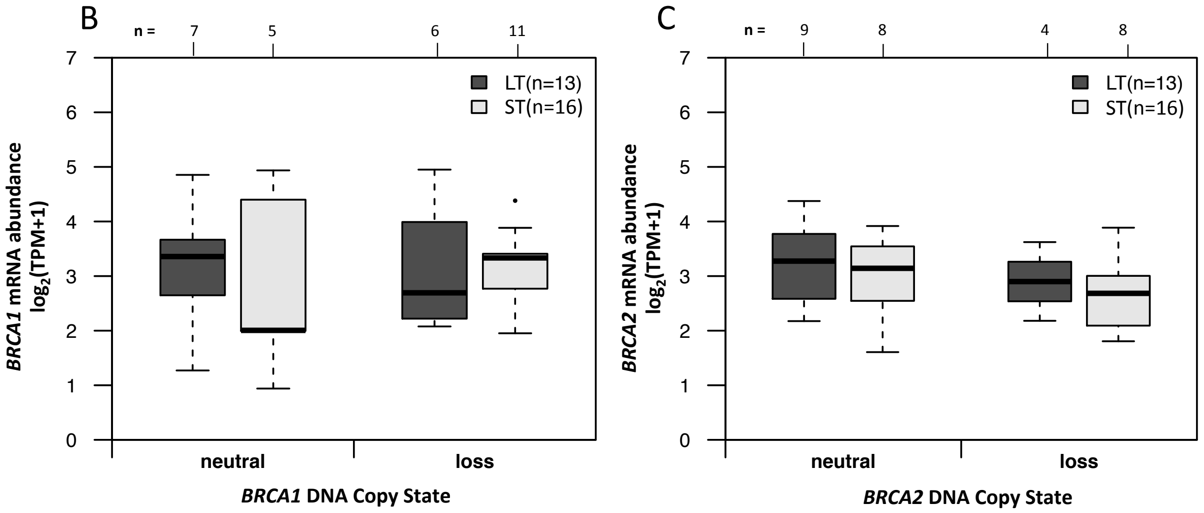

Figure S3. Copy state and gene-expression of *BRCA1* and *BRCA2.*

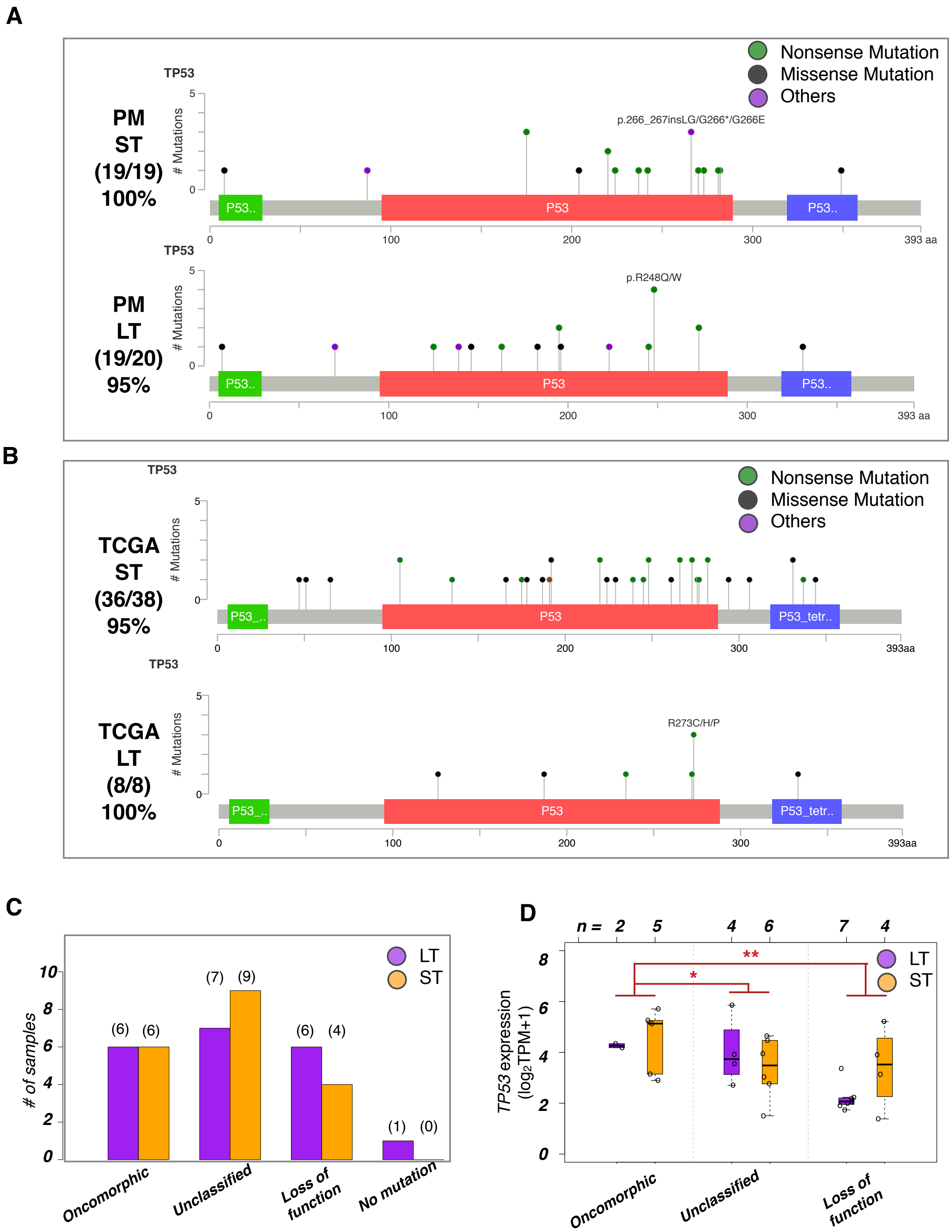

Figure S4. TP53 mutation comparison between LT and ST.

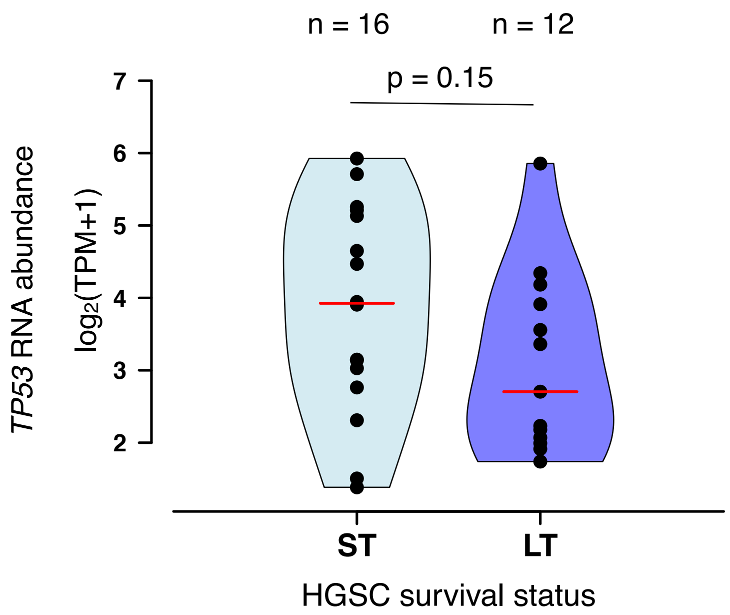

Figure S5. *TP53* transcript abundance comparison between short- and long-term survivor patients. Medians for each group are shown by the red horizontal line. Statistical significance was tested using two-sided Wilcoxon rank-sum test.

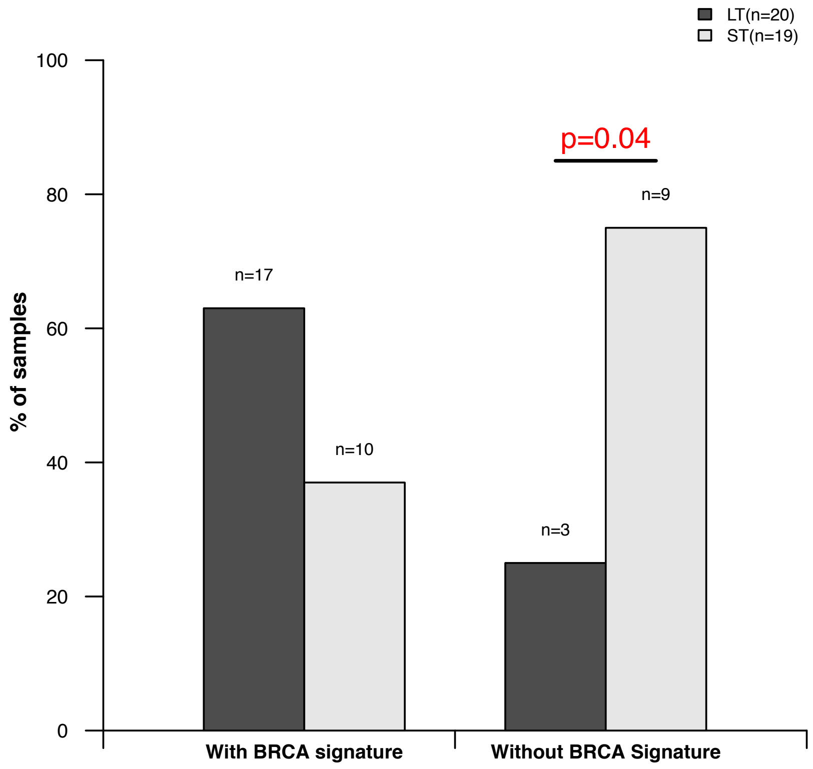

Figure S6. Mutation signature enrichment.

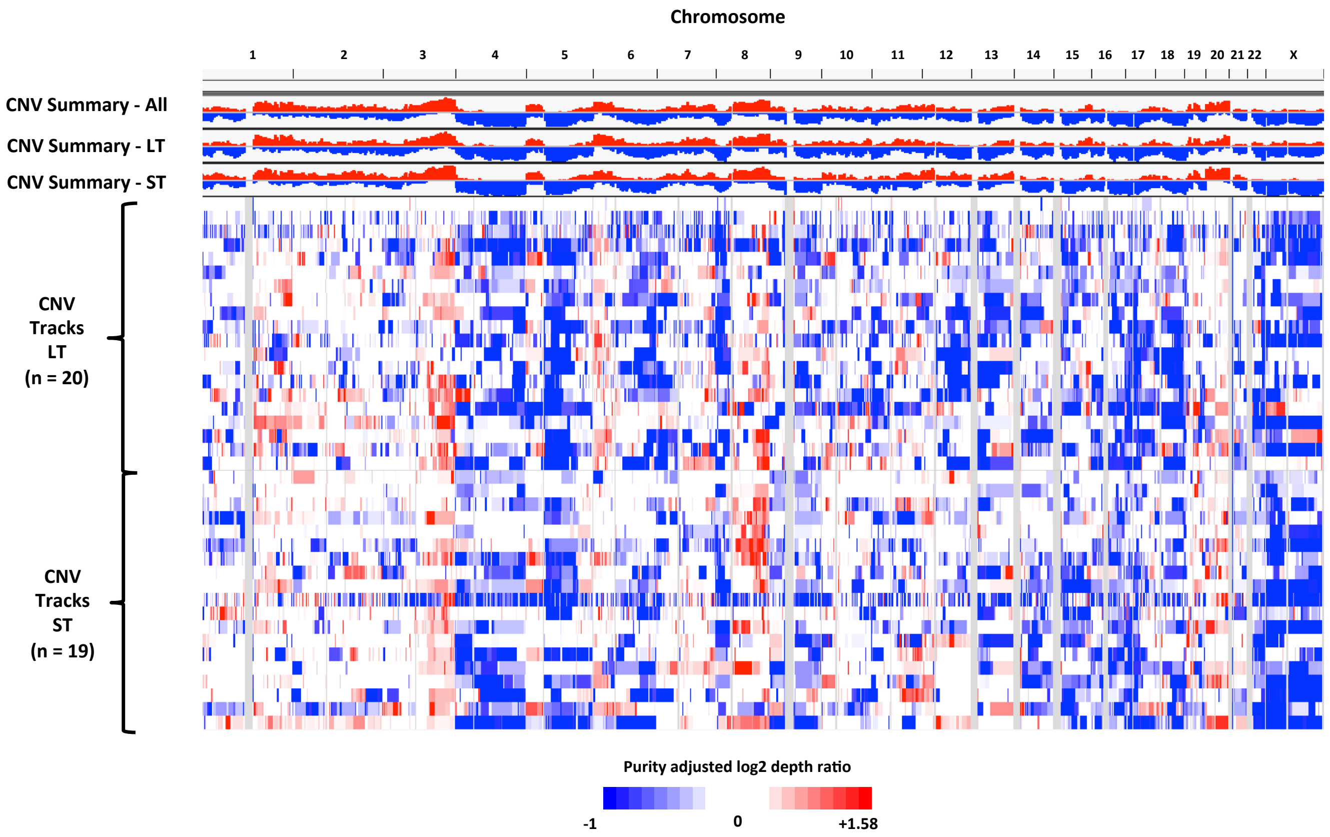

Figure S7. Copy number profile for all samples visualized in IGV. Blue indicates copy number loss while red indicates copy number gain.

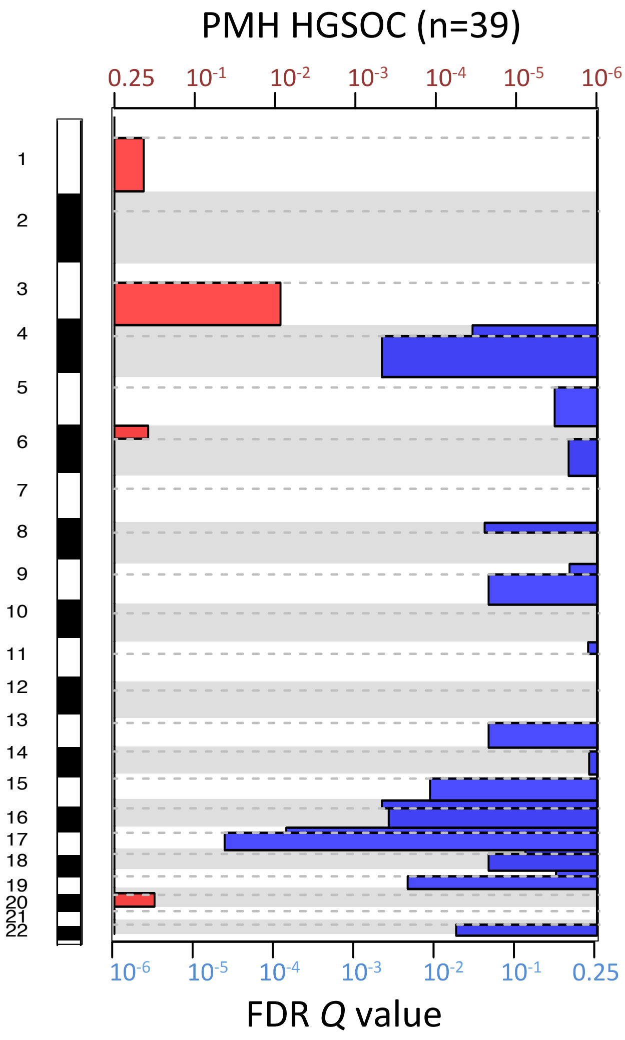

Figure S8. Arm-level copy number alterations in cohort.

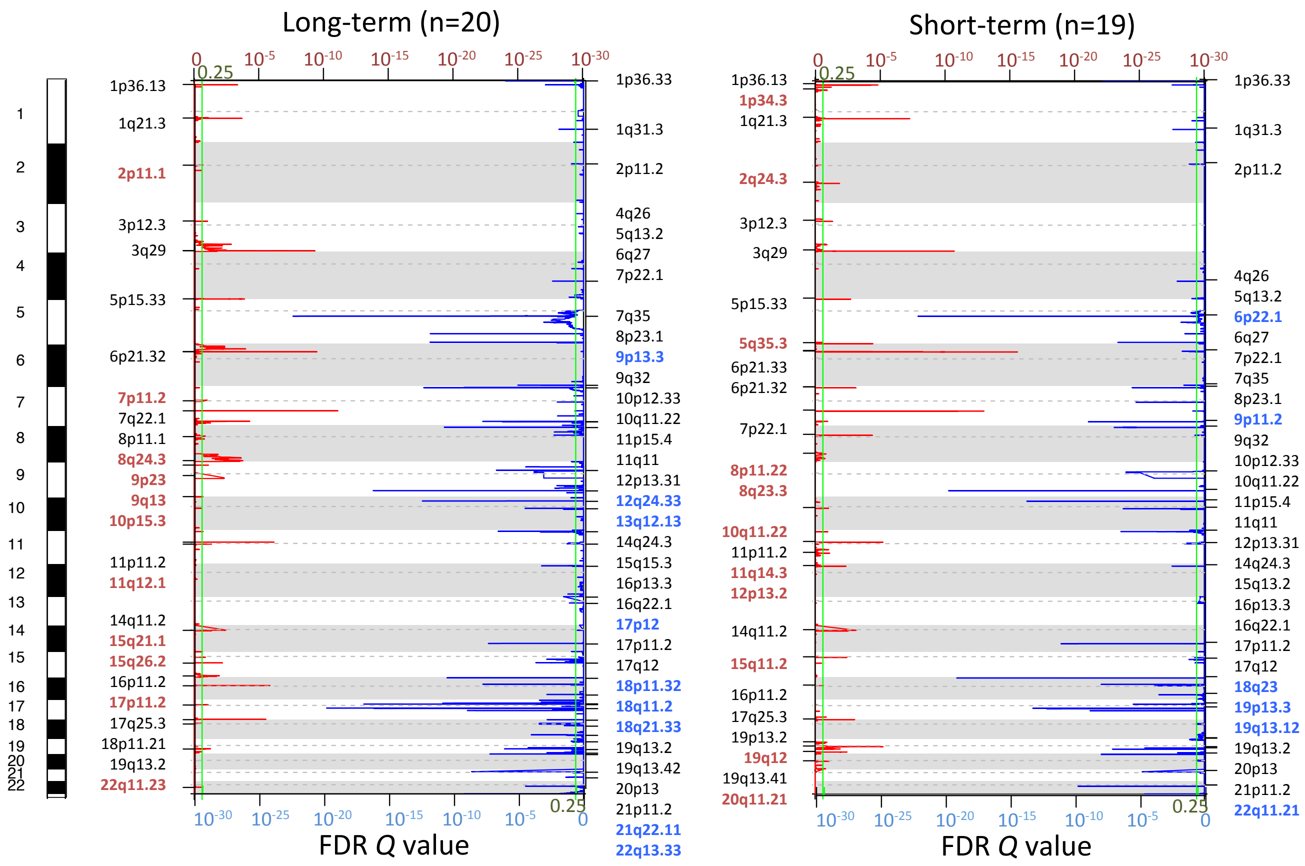

Figure S9. Focal copy number alterations in study cohort

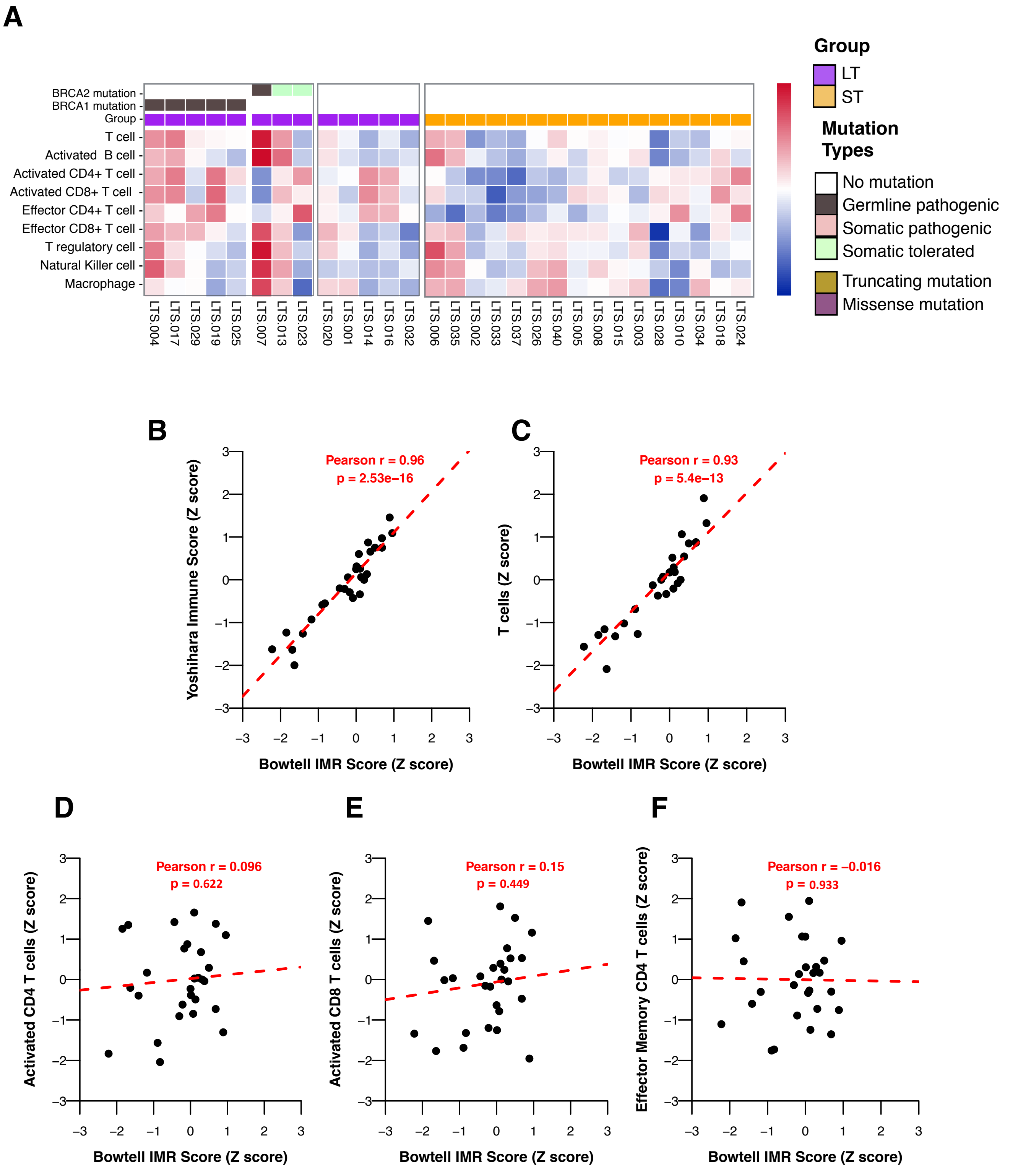

Figure S10. Immune score correlations.

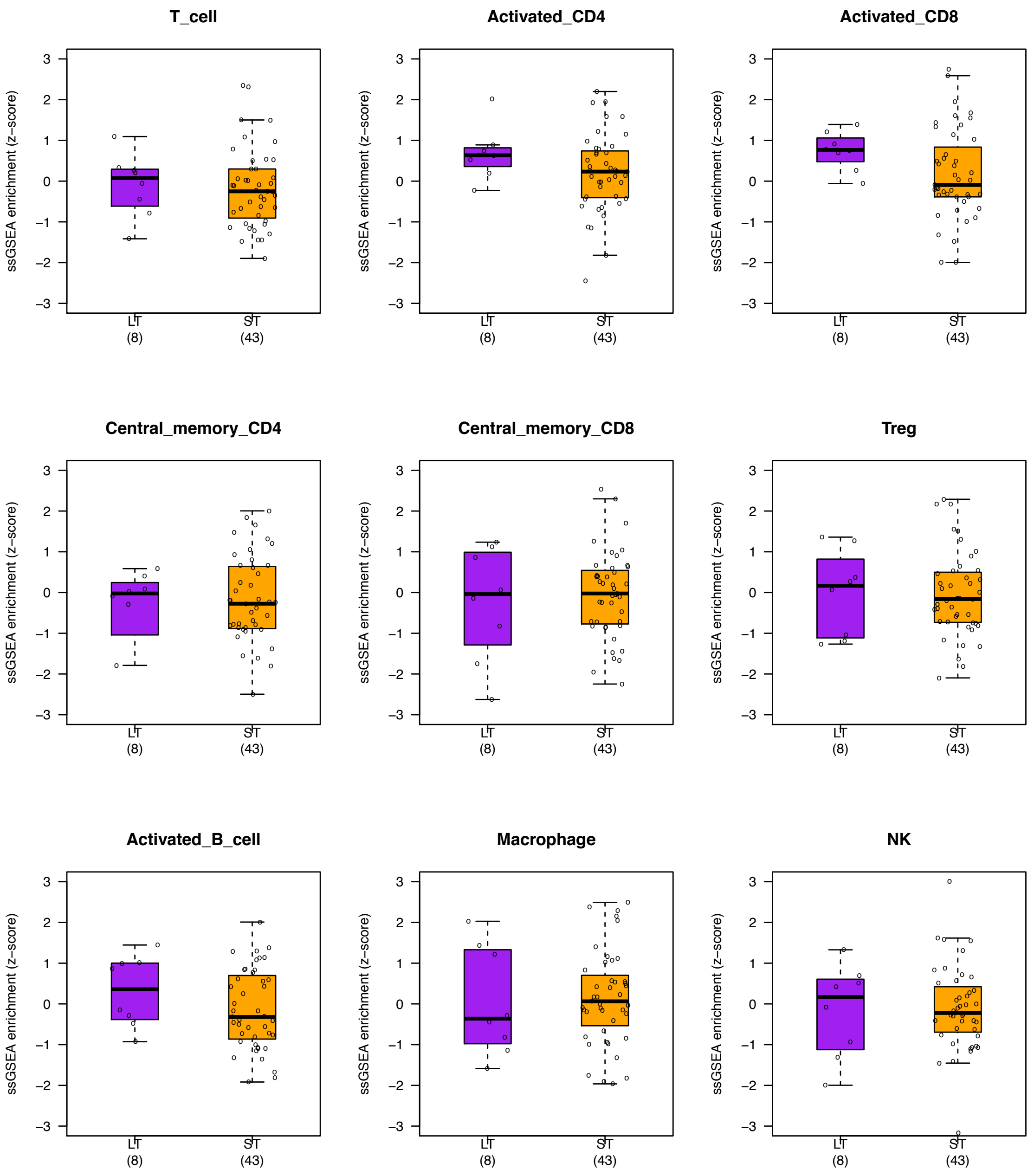

Figure S11. TCGA immune cell subtype enrichment comparison.

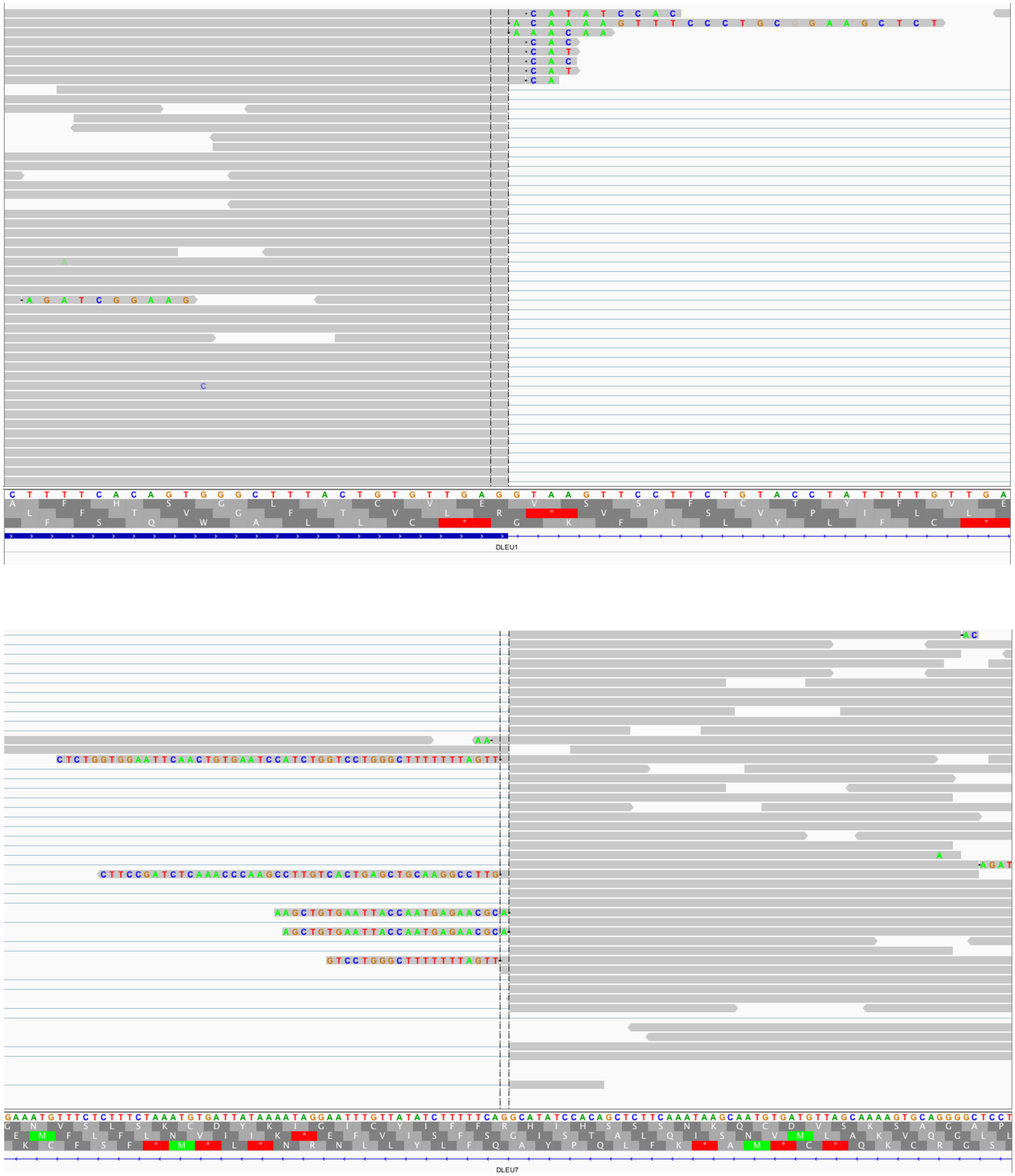

Figure S12. *DLEU1-DLEU7* in LTS-014

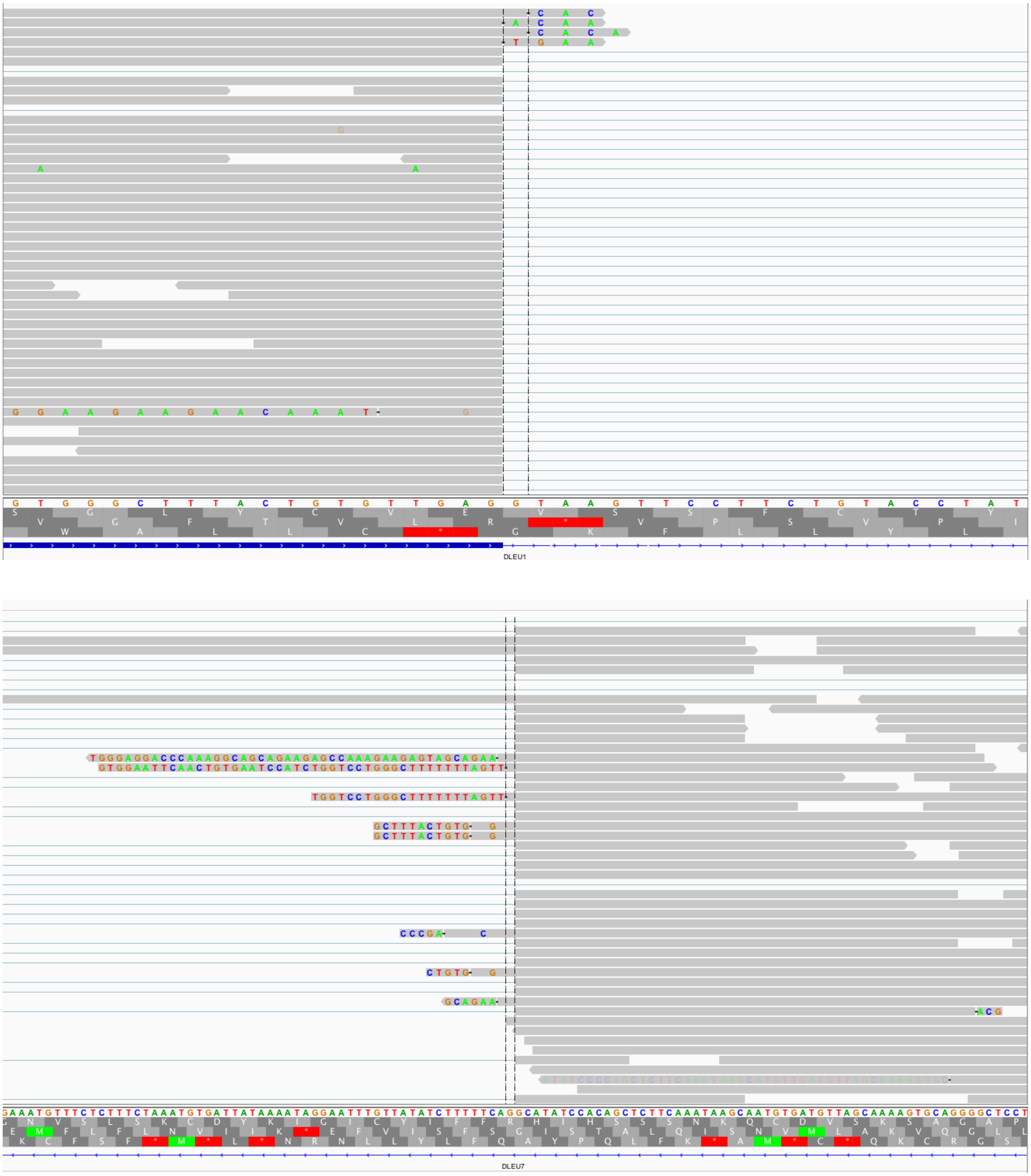

Figure S13.*DLEU1-DLEU7* in LTS-032

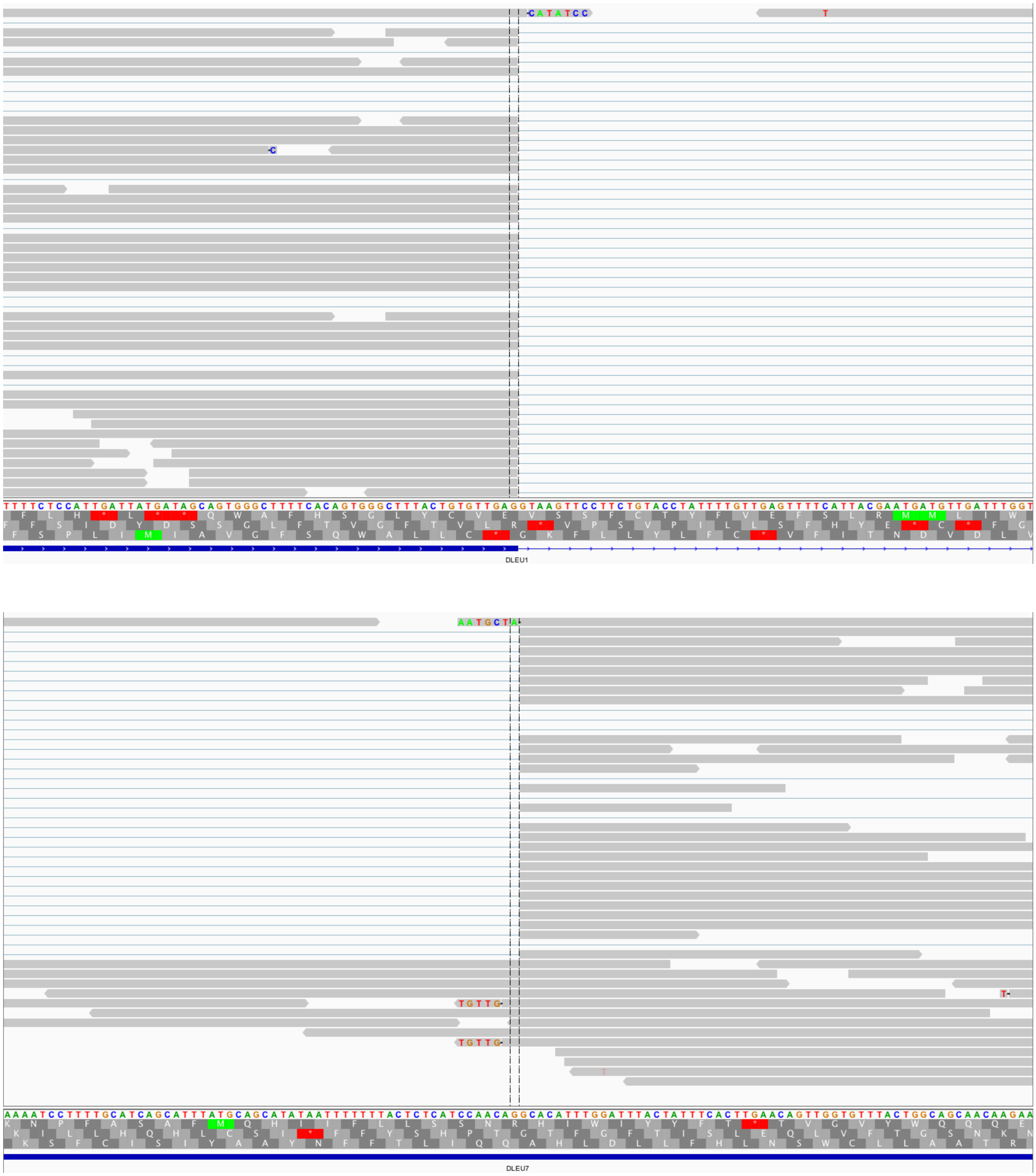

Figure S14. *DLEU1-DLEU7* in LTS-033

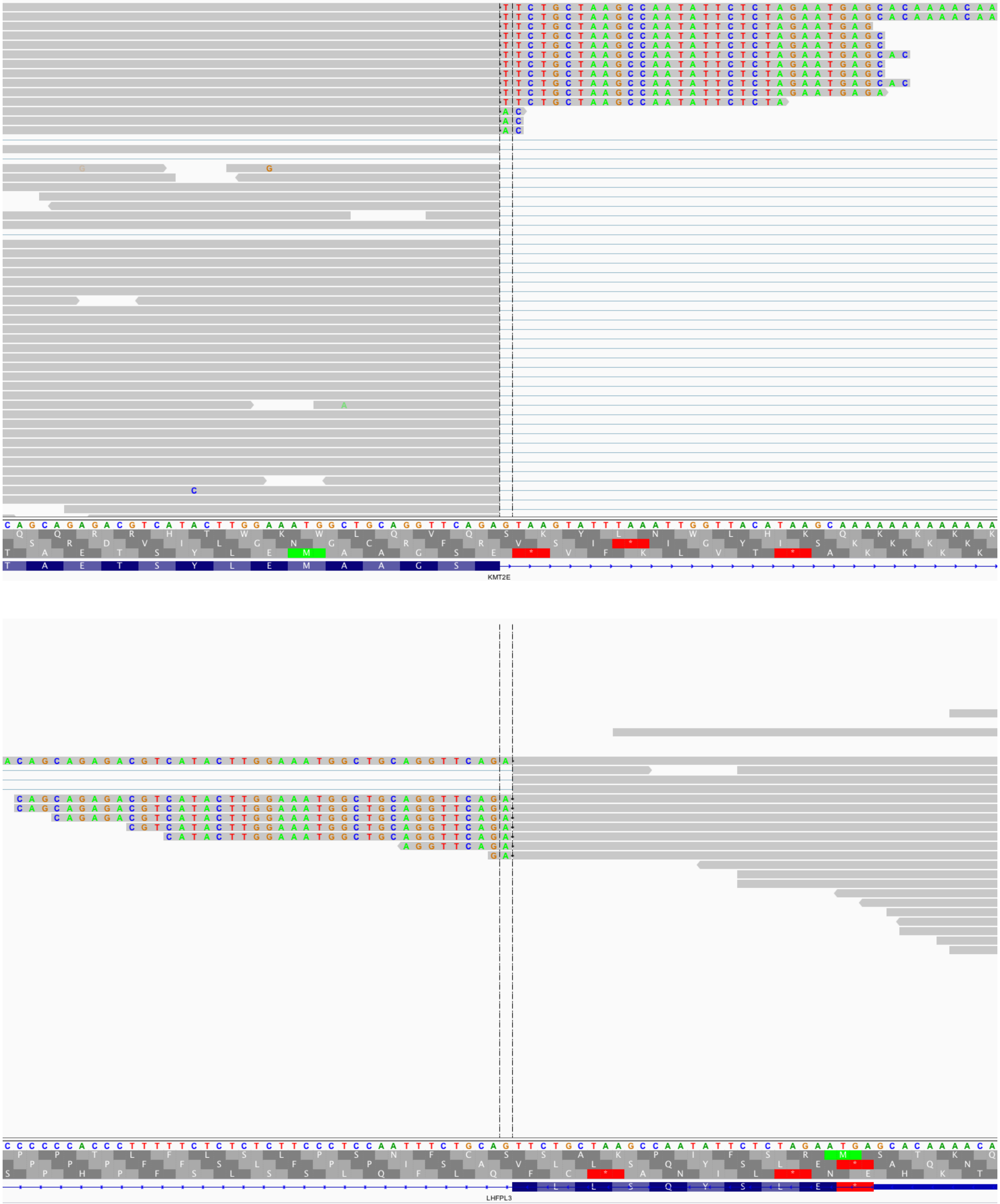

Figure S15. *KMT2E-LHFPL3* IN LTS-018

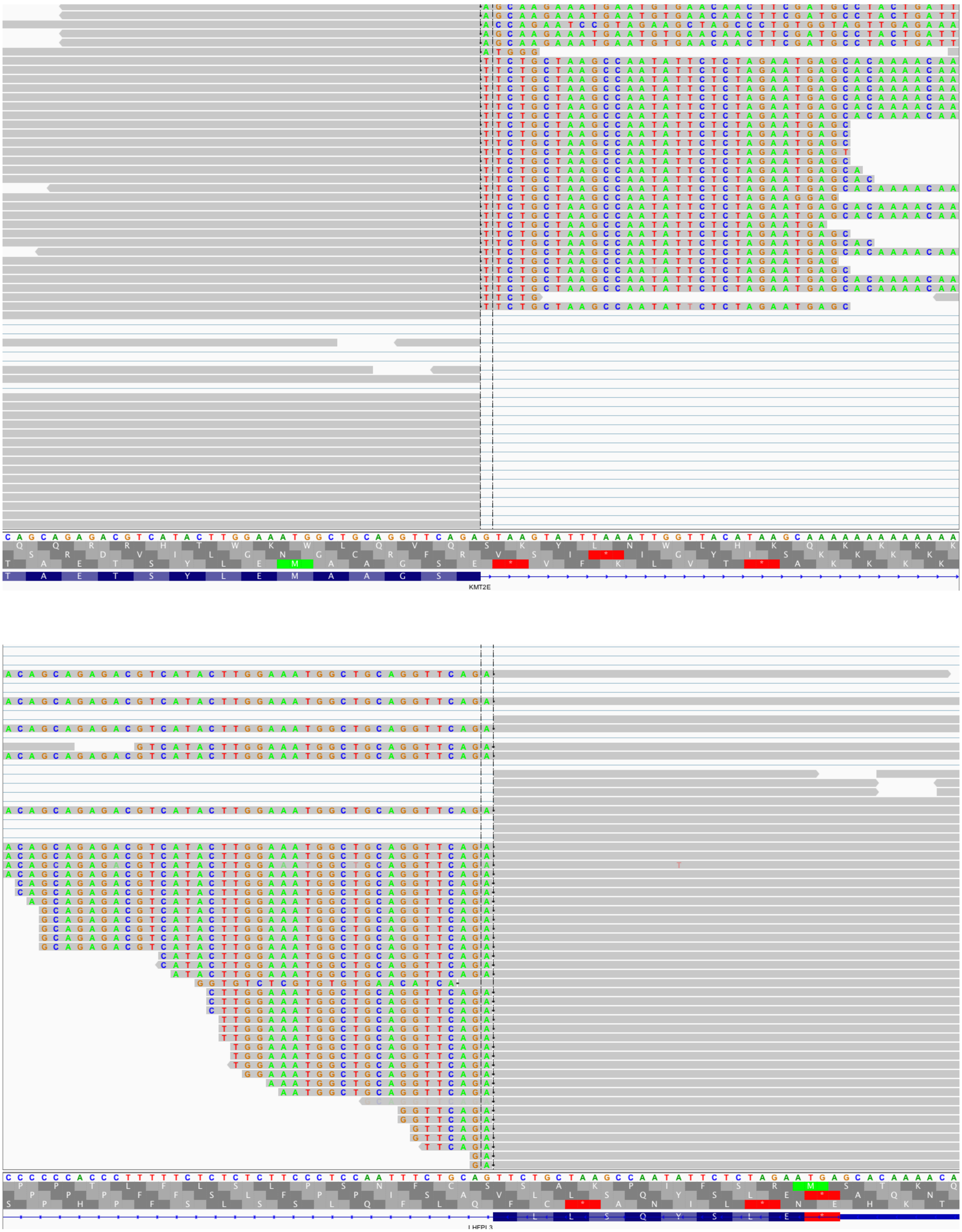

Figure S16. *KMT2E-LHFPL3* IN LTS-035

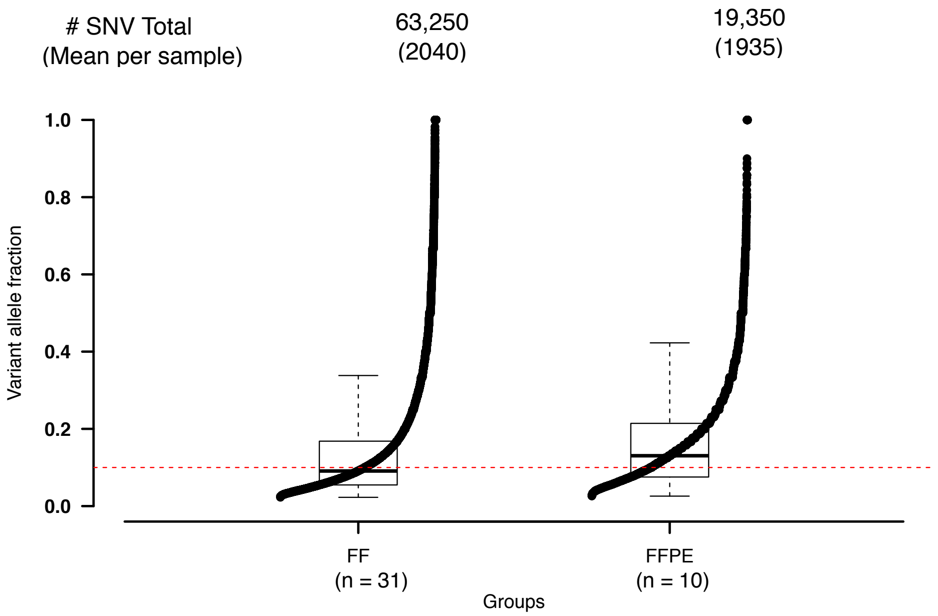

Figure S17. Distribution of all variant allele frequencies comparing fresh-frozen and formalin-fixed paraffin embedded samples. VAF cutoff of 0.1 used for analysis is indicated as the dotted horizontal red line. Total number and mean number of SNV per sample is annotated above each group.

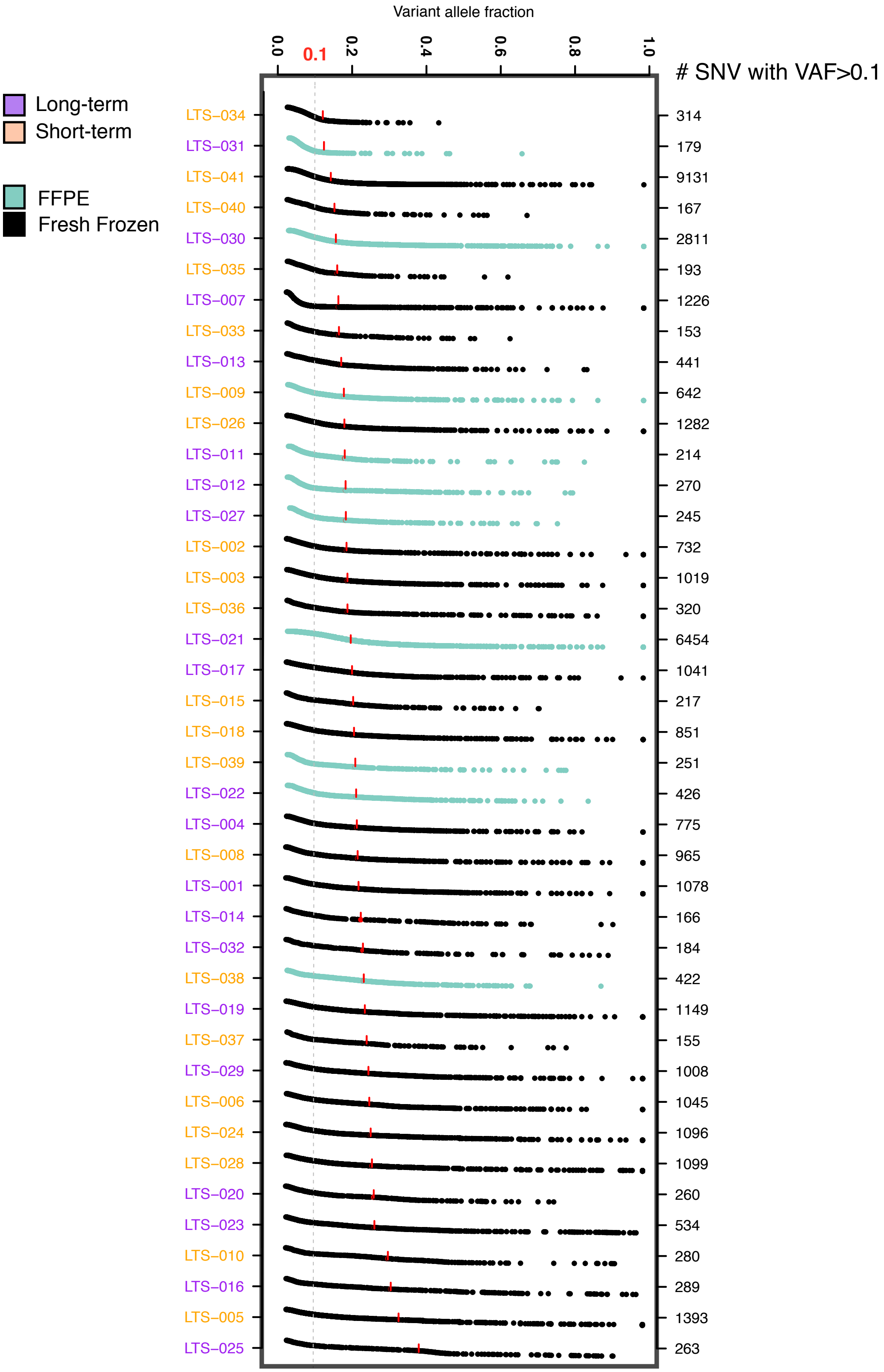

Figure S18. Distribution of variant allele frequencies for all samples with whole exome sequencing data. Samples are ordered by increasing order of median VAF and coloured according to sample preservation methods (FFPE -teal; Fresh Frozen -black). Survival groups are indicated as coloured sample IDs along the x-axis (Long-term- purple; Short-term-orange). Total number of SNV detected per sample with VAF > 0.1 indicated along the top axis.
