# Supplementary material for: Landscape of genomic alterations in high-grade serous ovarian cancer from exceptional long- and short-term survivors
Source: Genome Med. 2018 Oct 31;10:81. doi: 10.1186/s13073-018-0590-x (PMC6208125; doi:10.1186/s13073-018-0590-x)
Supplement: Supplementary file 2 — Supplementary methods for the manuscript. (PDF 112 kb) [file 13073_2018_590_MOESM2_ESM.pdf]

Additional file 2: Supplementary Methods

TCGA Data

To validate the findings from this study, we identified a subset of 42 (10 LT, 32 ST) HGSOC from TCGA that matched the clinical and survival description of our study cohort using the most complete patient information downloaded on November 1, 2016. Curated and annotated somatic mutations from WES sequencing and segmented copy ratio from Affymetrix 6.0 single nucleotide polymorphism (SNP) arrays for each sample in the TCGA ovarian cancer study were downloaded from Broad GDAC Firebrowse (http://firebrowse.org/?cohort=OV/). RNA-seq V2 FASTQ files for each TCGA OV sample was downloaded from Genomic Data Commons Data Portal (https://portal.gdc.cancer.gov) with controlled data access permissions and processed through the in-house RNA-seq data pre-processing pipeline as described below.

Whole Exome Analysis

Library Preparation and Sequencing: 200 ng from each DNA sample was used to generate libraries following Agilent SureSelect XT target enrichment kit as per protocol. 500ng to 750ng of amplified library from each sample was hybridized for 24 hours using baits from Agilent SureSelect Human All Exon V5 +UTRs. Size distribution of captured libraries was verified using an Agilent Bioanalyzer and concentration measured by qPCR. All libraries were normalized to 10nM and pooled. 12pM of pooled libraries were loaded onto Illumina cBot for cluster generation and the flow cell sequenced using 100bp paired-end reads using Illumina HiSeq 2000 or 2500 instruments. Tumor samples were sequenced to achieve 250X coverage and patient matched normal tissue DNA samples 50X coverage at the Princess Margaret Genomics Centre (PMGC).

Data Pre-processing: Following the Genome Analysis ToolKit (GATK) Best Practices for Somatic SNV Discovery in Whole Genome and Exome Sequence [1–3], raw sequencing reads were aligned to the human genome reference sequence (hg19) using the Burrows-Wheeler Alignment (bwa) tool (version

0.7.12) [4].The aligned BAM file was further processed to flag PCR duplicate reads using Picard MarkDuplicates tool (version 1.130). For each tumor and normal tissue data pair, regions with insertions and deletions (Indels) were realigned using GATK IndelRealigner (version 1.130) to minimize number of mismatched bases across all reads. To identify potential sample misannotation between patients, genotype correlation of germline single nucleotide polymorphisms (SNPs) detected using HaplotypeCaller (version 3.0) were compared between all samples sequenced in the study.

RNA-seq Analysis

Library Preparation and Sequencing: RNA samples were quantified by Qubit (Life Technologies) and quality by Agilent Bioananlyzer. All samples with RNA integrity (RIN) score above 8 were considered intact. Libraries were prepared using TruSeq Stranded Total RNA kit (Illumina). One hundred fifty nanograms from tumor RNA samples were ribosomal RNA depleted using Ribo-zero Gold rRNA beads, following purification intact RNA was fragmented. RNA samples with Bioananlyzer RIN score between 1-2.8 were not fragmented. The cleaved RNA fragments were copied into first strand cDNA using reverse transcriptase and random primers. This is followed by second strand cDNA synthesis using RNase H and DNA Polymerase I. A single “A” based were added and adapter ligated followed by purification and enrichment with PCR to create cDNA libraries. Final cDNA libraries were size validated using Agilent Bioanalyzer and concentration validated by qPCR. All libraries were normalized to 10nM and pooled together. 11pM of pooled libraries were loaded onto Illumina cBot for cluster generation. Clustered flow cell was then sequenced pair-end 100 cycles V3 using Illumina HiSeq 2000 to achieve a minimum of ~80 million reads per sample at the PMGC.

Data Pre-processing: FASTQ files containing paired-end raw reads are aligned to human genome reference GRCh37 with transcript annotation GENCODE v19 using STAR (version 2.4.2a) [5] two-pass method. Gene-wise transcript abundances are quantified using RSEM (version 1.2.29) [6] into units of transcripts per million (TPM). RNA-seq data quality for each sample was assessed based on total number of unique read fragments sampled (estimated # unique reads). RNA-seq data from all

FFPE preserved tumor samples were excluded from whole transcriptome analysis due to low data quality.

TP53 Targeted Deep Sequencing

Library Preparation and Sequencing:

Sequencing libraries were prepared for 7 DNA samples from tumors lacking detectable *TP53* mutation by exome sequencing. DNA samples were first sheared to 300ng, and 100ng of DNA was used to generate libraries using the KAPA Hyper Prep Kit protocol v5.16. Adapter ligation was completed overnight using NEXTflex adapters and 5 cycles were completed in library amplification. Library DNA samples were pooled by sample type: FFPE DNA and Fresh Frozen (FF) DNA. 143ng of each FF library DNA sample were pooled together for a total of ~1000ng capture sample. 100-110ng of each FFPE library DNA were pooled together for a total of 630ng capture sample. Target capture was completed using the IDT Hybridization capture of DNA libraries using custom xGen Lockdown Probes (covering all exons of *TP53*, *KRAS*, *CDKN2A*, and *SMAD4*) and Reagents v.2 protocol. 0.616pmol of custom designed capture probes was added to each capture, followed by overnight hybridization (~15 hours). Post capture PCR enrichment for FFPE and FF captures was set to 16 cycles. Libraries were pooled and loaded onto Illumina cBot for cluster generation and the flow cell sequenced using V4 125bp paired-end reads using Illumina HiSeq 2500 to a total of 40 million reads at the PMGC.

DNA Mutation Analysis

Germline mutations in known DNA repair genes in ovarian cancer (*BRCA1*, *BRCA2 RAD51*, *RAD51C*, *RAD51D*) were identified using GATK HaplotypeCaller (version 1.130) from normal tissue BAM files with default settings. Germ-line mutation calls were annotated using Oncotator (version 1.5.3) [7]. Variants in DNA repair genes were matched to variants described in the NCBI ClinVar Database (version 12.03.20) and prioritized according to reported clinical significance.

The complete somatic sequence mutation profile for each tumor sample is generated using MuTect (version 1.1.4) for single nucleotide variations (SNVs) and Strelka (version 1.0.14) [8] for small insertions and deletions (Indels) on paired normal and tumor tissue BAM files. Variants are annotated using Oncotator (version 1.5.3). Variants with low variant allelic frequency (VAF < 0.10) are excluded from subsequent analysis in concordance with the variant detection threshold used in the TCGA OV study. Wong *et al.* [9] have demonstrated, by conducting deep targeted sequencing of DNA from formalin treated samples, that the majority of the formalin-treatment induced single nucleotide changes occur at allele frequency of less than 10%. By applying this threshold, we did not detect any statistical significant difference in the total number of SNVs detected in formalin-treated compared to untreated samples (Additional file: Fig. S17). In addition, although more formalin-treated samples were included within the long-term survivors cohort as compare to the short-term survivors group, we did not observe an association between mutation burden and formalin-treatment that would explain our mutation burden finding between the two survival groups (Additional file 1: Fig. S18). Recurrently mutated genes by SNVs and Indels were identified separately for each survival cohort using MutSigCV (version 1.4) [10] from Oncotator output.

As a denominator for calculating mutation frequency, we defined a bed file describing genomic intervals for mutation calling from all overlapping regions between coding exon regions (Human genome reference GRCh37 RefGene UCSC Track) with 2 base pair padding at start and end with genomic intervals targeted by SureSelect Human All Exons + UTR V5 probe design. Mutations were identified using MuTect (version 1.1.4) from paired normal and tumor exome sequence bam files. To calculate mutation rate, the denominator describes the total number of bases with sufficient coverage (7 in normal and 14 in tumor) for mutation call by MuTect within coding exonic intervals +/- 2 bases (mean 30 Mbp). The numerator contains all non-synonymous somatic mutations detected the covered coding genomic regions with minor allele frequency exceeding 5%.

Composition of previously defined mutational signatures for each sample was inferred from somatic SNVs with variant allele frequency greater than 10% using the DeconstructSigs R-package (version 1.8.0) [11]. By default, all scores greater than 2% were reported in the analysis output.

Power Analysis

We conducted power analysis for our study. Considering that this study is exploratory and hypothesis generating, all statistical tests will be two-sided with the significance level defined as 0.05. Give the 20 LT and 21 ST subjects, assuming a strong effect (mean difference between LT and ST groups) to be 1.2 standard deviation (SD) of the biomarker, with two-sided significance level at 0.05, our study will provide 96% power to detect statistically significant association. However, if assuming a moderate effect (mean difference between LT and ST groups) to be 1.0 SD of the biomarker, with two-sided significance level at 0.05, our study will provide 87% power to detect statistically significant association. For a weak effect with 0.5 SD of the biomarker, our study will have very low power (35%) to detect statistically significant association.

References

1. DePristo MA, Banks E, Poplin R, Garimella KV, Maguire JR, Hartl C, et al. A framework for variation discovery and genotyping using next-generation DNA sequencing data. Nat Genet. 2011;43:491–8.

2. McKenna A, Hanna M, Banks E, Sivachenko A, Cibulskis K, Kernytsky A, et al. The Genome Analysis Toolkit: a MapReduce framework for analyzing next-generation DNA sequencing data. Genome Res. 2010;20:1297–303.

3. Van der Auwera GA, Carneiro MO, Hartl C, Poplin R, Del Angel G, Levy-Moonshine A, et al. From FastQ data to high confidence variant calls: the Genome Analysis Toolkit best practices pipeline. Curr Protoc Bioinforma. 2013;43:11.10.1–33.

4. Li H, Durbin R. Fast and accurate long-read alignment with Burrows-Wheeler transform. Bioinforma Oxf Engl. 2010;26:589–95.

5. Dobin A, Davis CA, Schlesinger F, Drenkow J, Zaleski C, Jha S, et al. STAR: ultrafast universal RNA-seq aligner. Bioinforma Oxf Engl. 2013;29:15–21.

6. Li B, Dewey CN. RSEM: accurate transcript quantification from RNA-Seq data with or without a reference genome. BMC Bioinformatics. 2011;12:323.

7. Ramos AH, Lichtenstein L, Gupta M, Lawrence MS, Pugh TJ, Saksena G, et al. Oncotator: cancer variant annotation tool. Hum Mutat. 2015;36:E2423–9.

8. Saunders CT, Wong WSW, Swamy S, Becq J, Murray LJ, Cheetham RK. Strelka: accurate somatic small-variant calling from sequenced tumor-normal sample pairs. Bioinforma Oxf Engl. 2012;28:1811–7.

9. Wong SQ, Li J, Tan AY-C, Vedururu R, Pang J-MB, Do H, et al. Sequence artefacts in a prospective series of formalin-fixed tumours tested for mutations in hotspot regions by massively parallel sequencing. BMC Med Genomics. 2014;7:23.

10. Lawrence MS, Stojanov P, Polak P, Kryukov GV, Cibulskis K, Sivachenko A, et al. Mutational heterogeneity in cancer and the search for new cancer-associated genes. Nature. 2013;499:214–8.

11. Rosenthal R, McGranahan N, Herrero J, Taylor BS, Swanton C. deconstructSigs: delineating mutational processes in single tumors distinguishes DNA repair deficiencies and patterns of carcinoma evolution. Genome Biol [Internet]. 2016 [cited 2017 Jun 19];17:31. Available from: http://dx.doi.org/10.1186/s13059-016-0893-4
