# Supplementary material for: Landscape of genomic alterations in high-grade serous ovarian cancer from exceptional long- and short-term survivors
Source: Genome Med. 2018 Oct 31;10:81. doi: 10.1186/s13073-018-0590-x (PMC6208125; doi:10.1186/s13073-018-0590-x)
Supplement: Supplementary file 3 — CNV segment size as distribution per sample. Distribution of CNV segment size as percentage of chromosome arm in each sequenced tumor sample. Sequenza estimated sample ploidy and the threshold used for determining copy number amplification is shown for each sample as colored horizontal lines. (PDF 34 kb) [file 13073_2018_590_MOESM3_ESM.pdf]

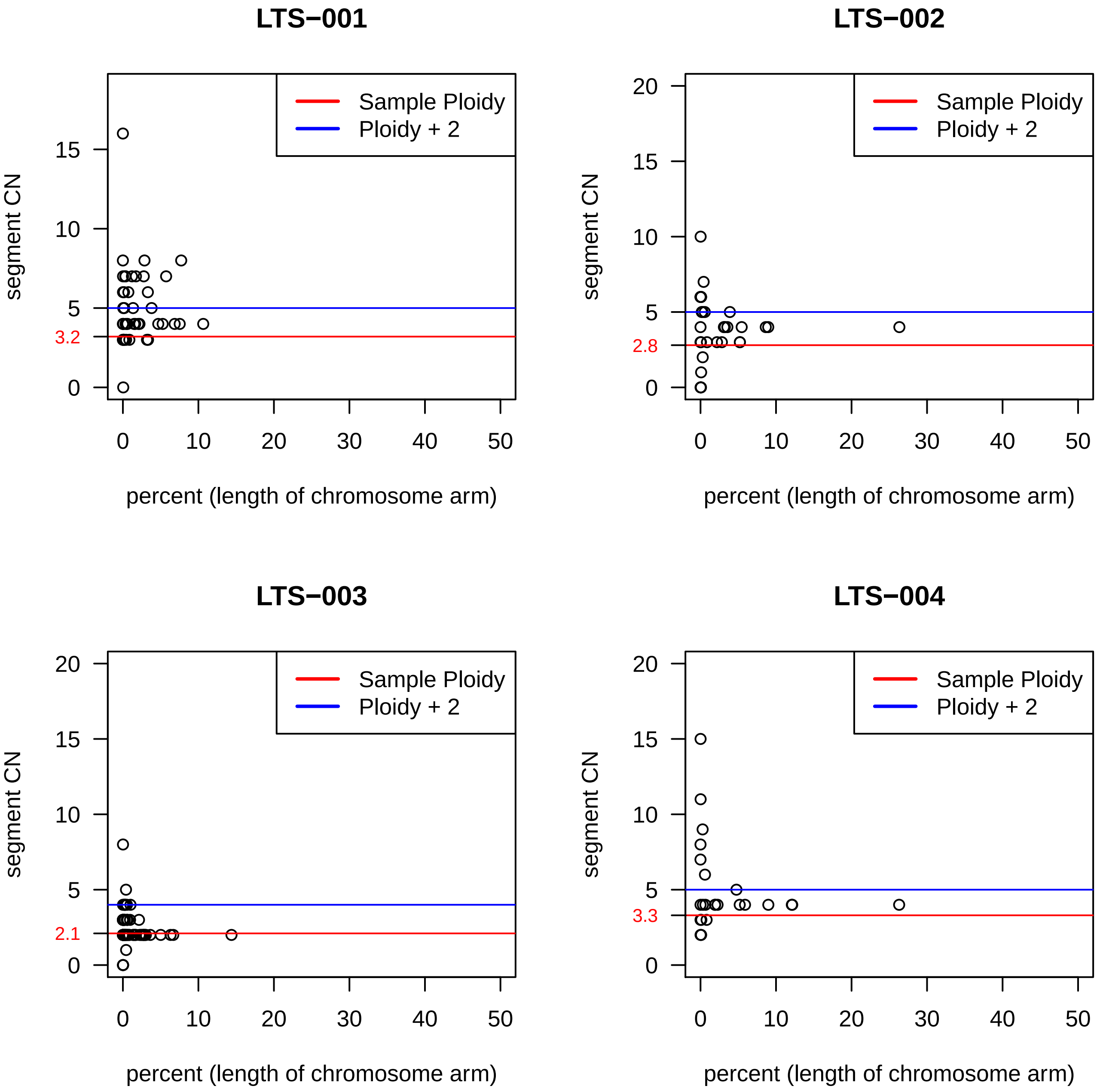
LTS−001
LTS−002
LTS−003
LTS−004
Sample Ploidy
Ploidy + 2
segment CN
percent (length of chromosome arm)
0
5
10
15
20
30
40
50
3.2
2.8
2.1
3.3

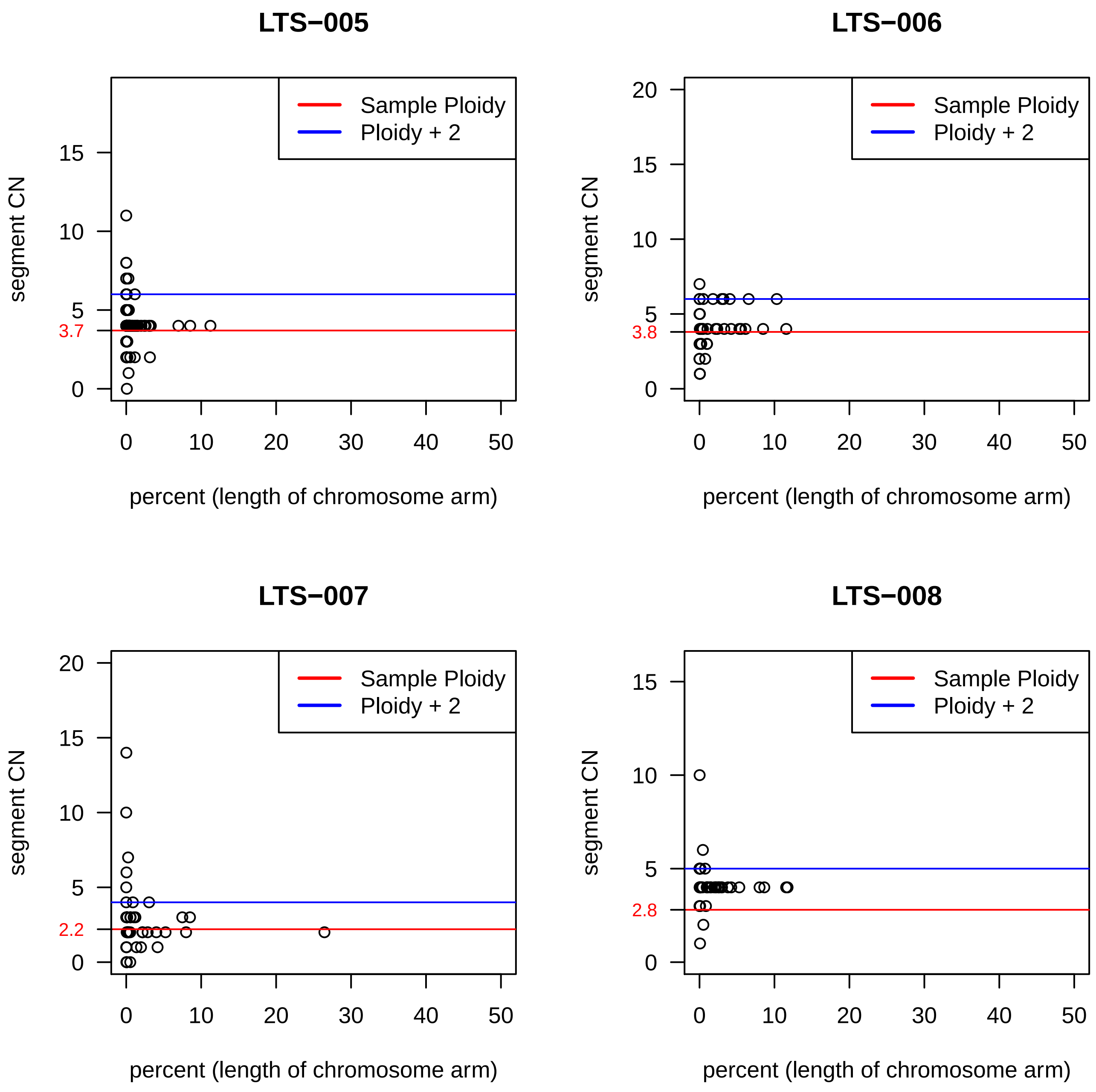

LTS−005
Sample Ploidy
Ploidy + 2
segment CN
percent (length of chromosome arm)
0
5
10
15
3.7
0
10
20
30
40
50
LTS−006
Sample Ploidy
Ploidy + 2
segment CN
percent (length of chromosome arm)
0
5
10
15
20
3.8
LTS−007
Sample Ploidy
Ploidy + 2
segment CN
percent (length of chromosome arm)
0
5
10
15
20
2.2
LTS−008
Sample Ploidy
Ploidy + 2
segment CN
percent (length of chromosome arm)
0
5
10
15
2.8

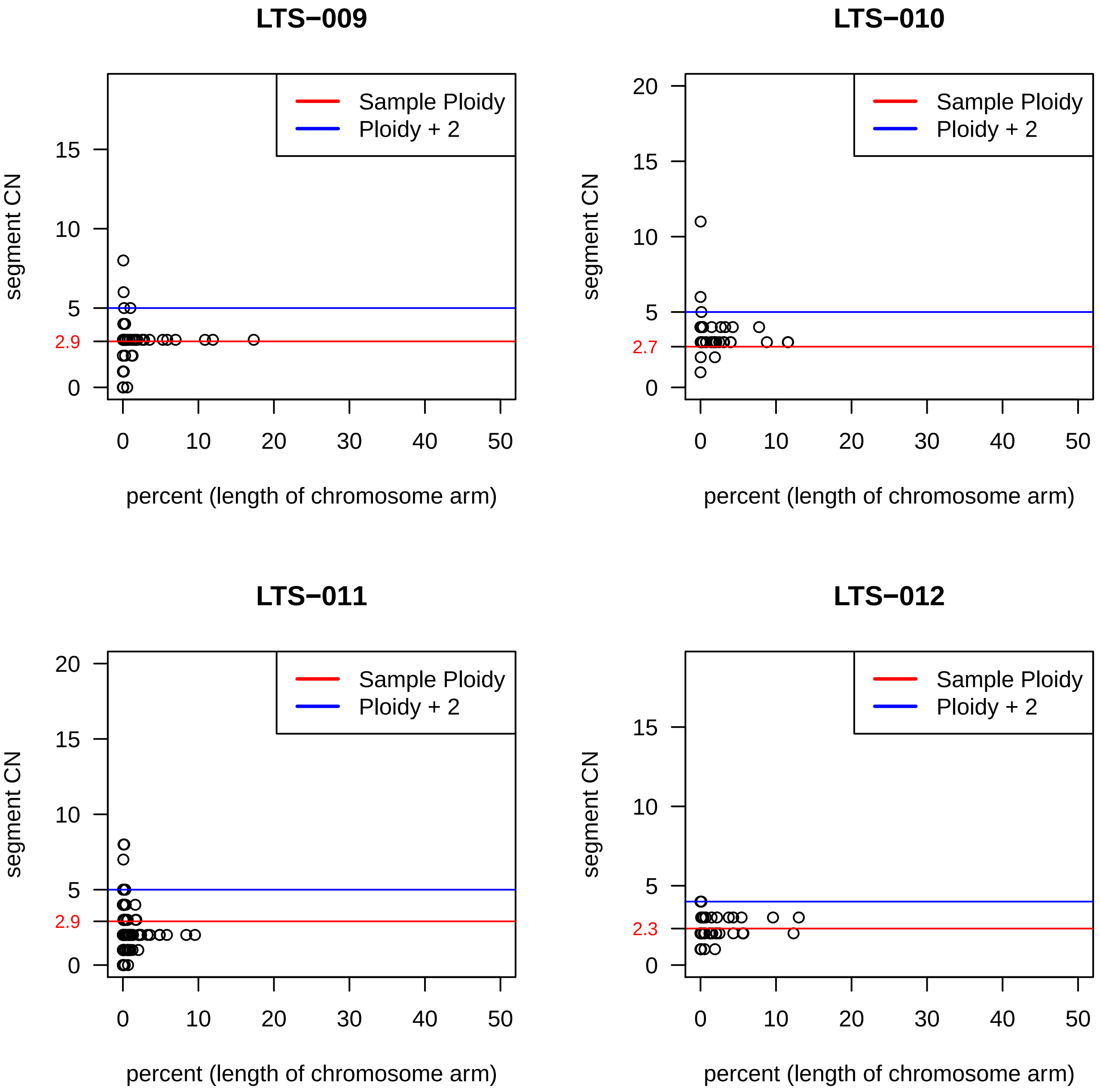
LTS−009
LTS−010
LTS−011
LTS−012
Sample Ploidy
Ploidy + 2
segment CN
percent (length of chromosome arm)
2.9
2.7
2.9
2.3

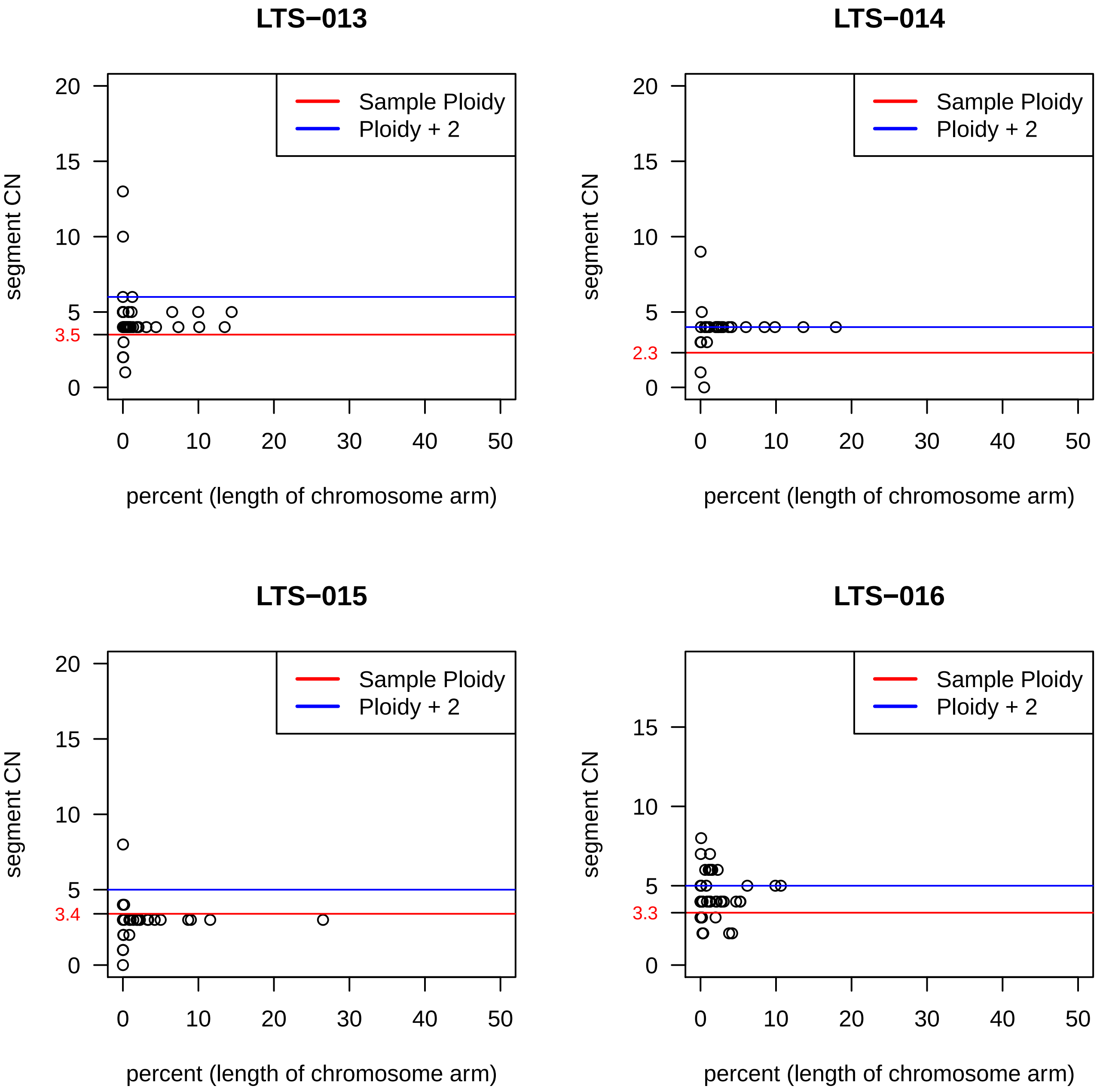

LTS−013
LTS−014
LTS−015
LTS−016
Sample Ploidy
Ploidy + 2
segment CN
percent (length of chromosome arm)
0
5
10
15
20
3.5
2.3
3.4
3.3
30
40
50

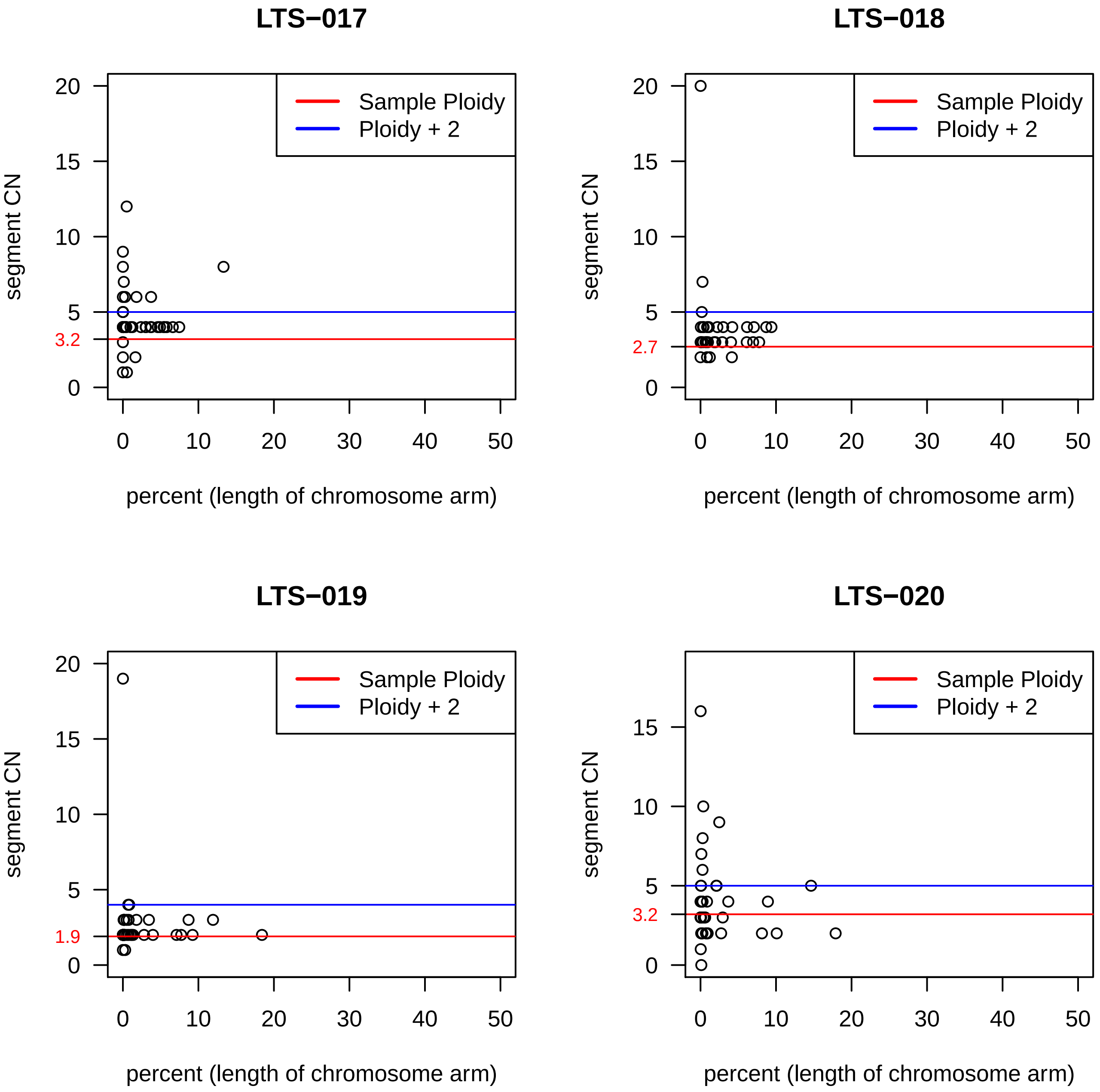
LTS−017
LTS−018
LTS−019
LTS−020
Sample Ploidy
Ploidy + 2
segment CN
percent (length of chromosome arm)
3.2
2.7
1.9
0
5
10
15
20
30
40
50

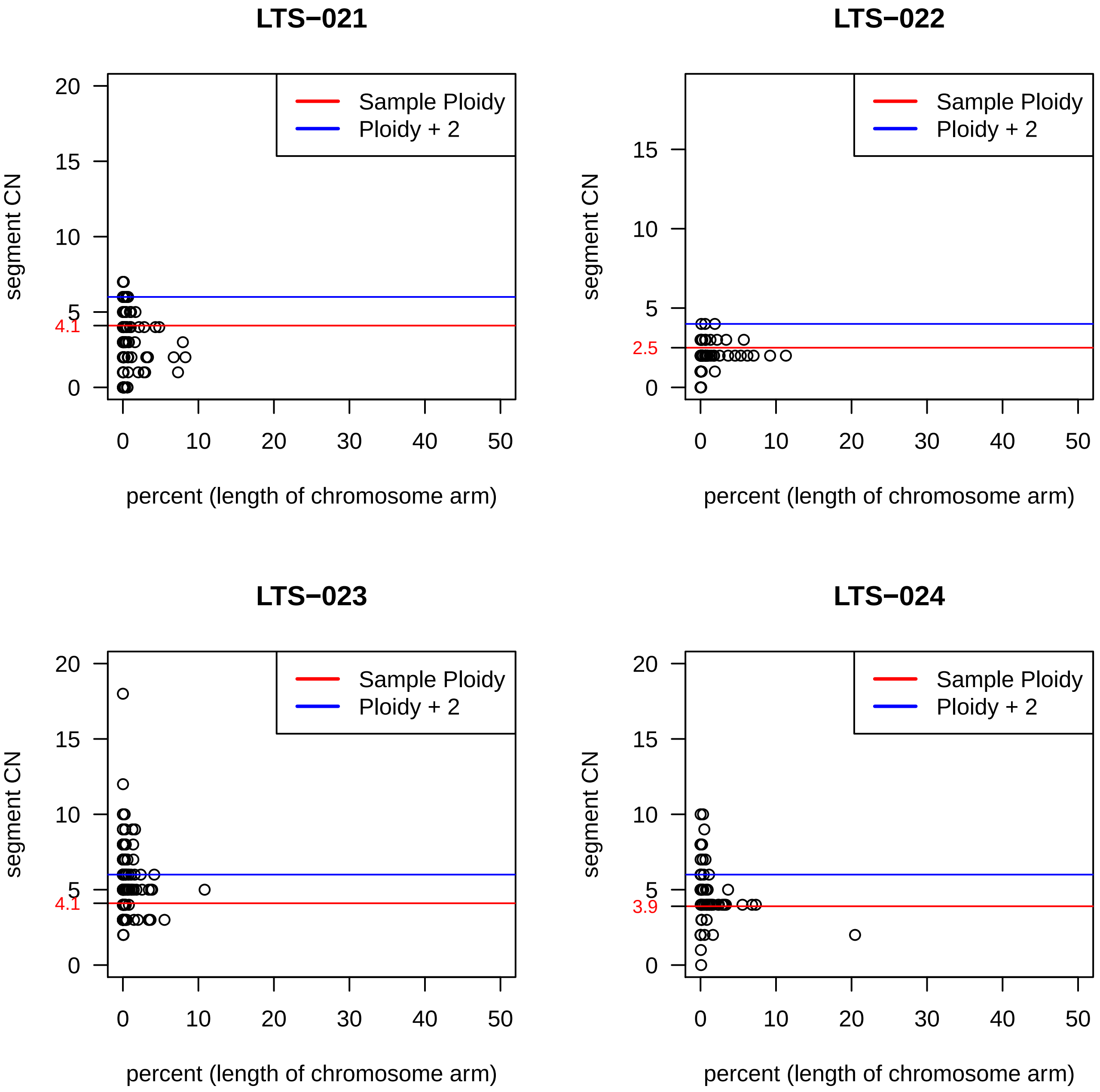
LTS−021
LTS−022
LTS−023
LTS−024
Sample Ploidy
Ploidy + 2
segment CN
percent (length of chromosome arm)
0
5
10
15
20
4.1
2.5
3.9
0
10
20
30
40
50

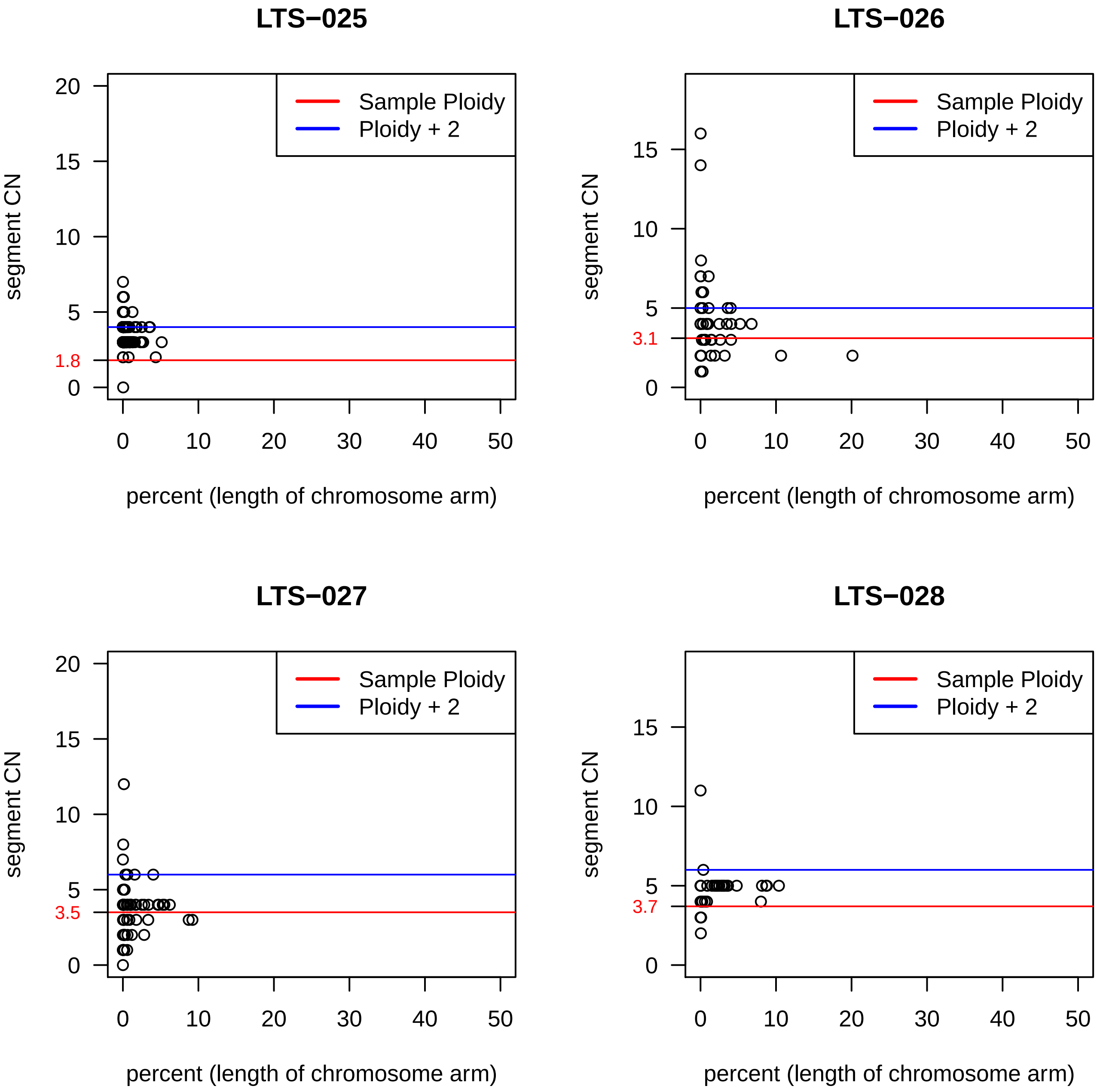
LTS−025
LTS−026
LTS−027
LTS−028
Sample Ploidy
Ploidy + 2
segment CN
percent (length of chromosome arm)
0
5
10
15
20
1.8
3.1
3.5
3.7
0
10
20
30
40
50

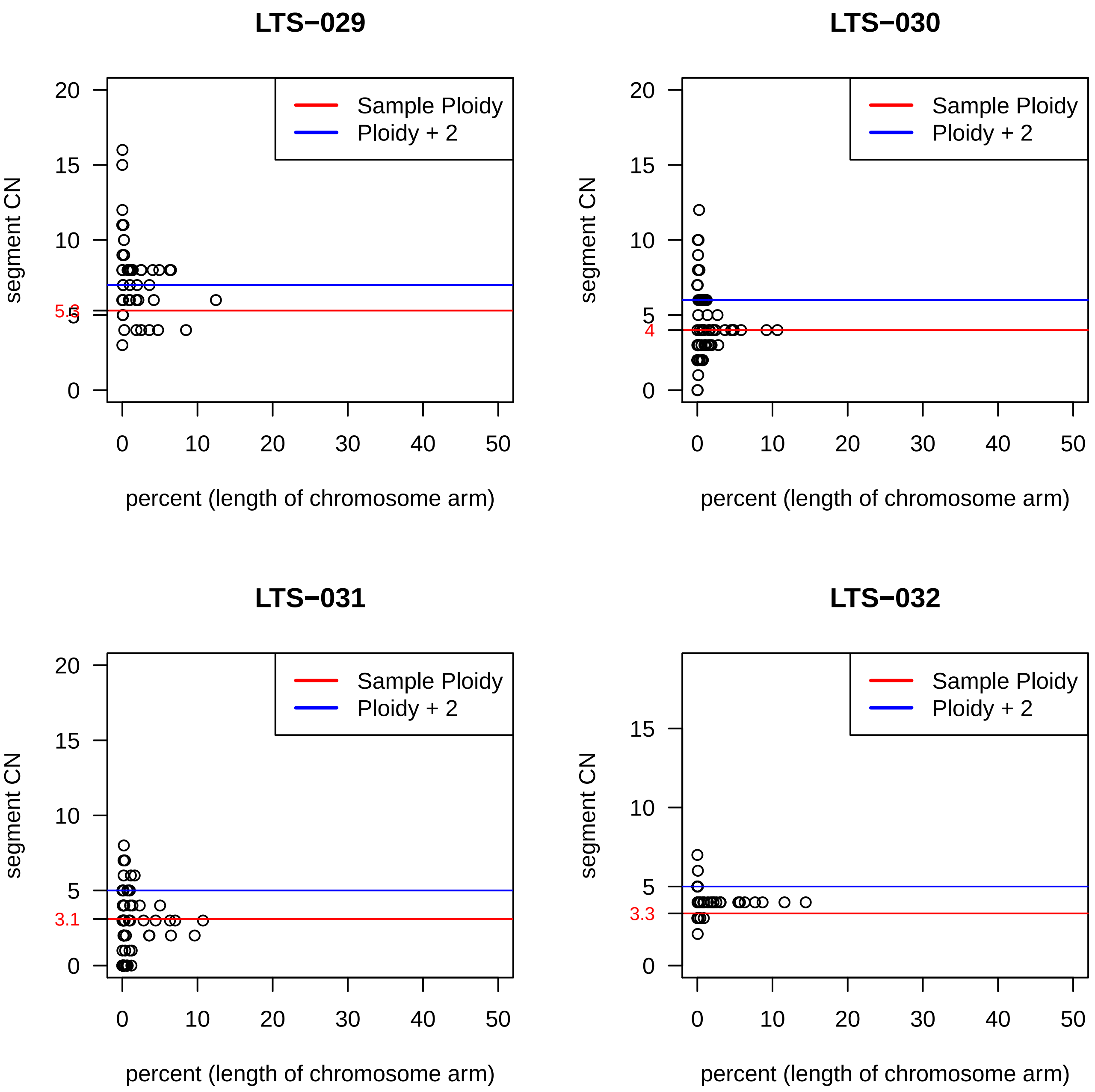
LTS−029
LTS−030
LTS−031
LTS−032
Sample Ploidy
Ploidy + 2
segment CN
percent (length of chromosome arm)
5.3
4
3.1
3.3
0
5
10
15
20
0
10
20
30
40
50

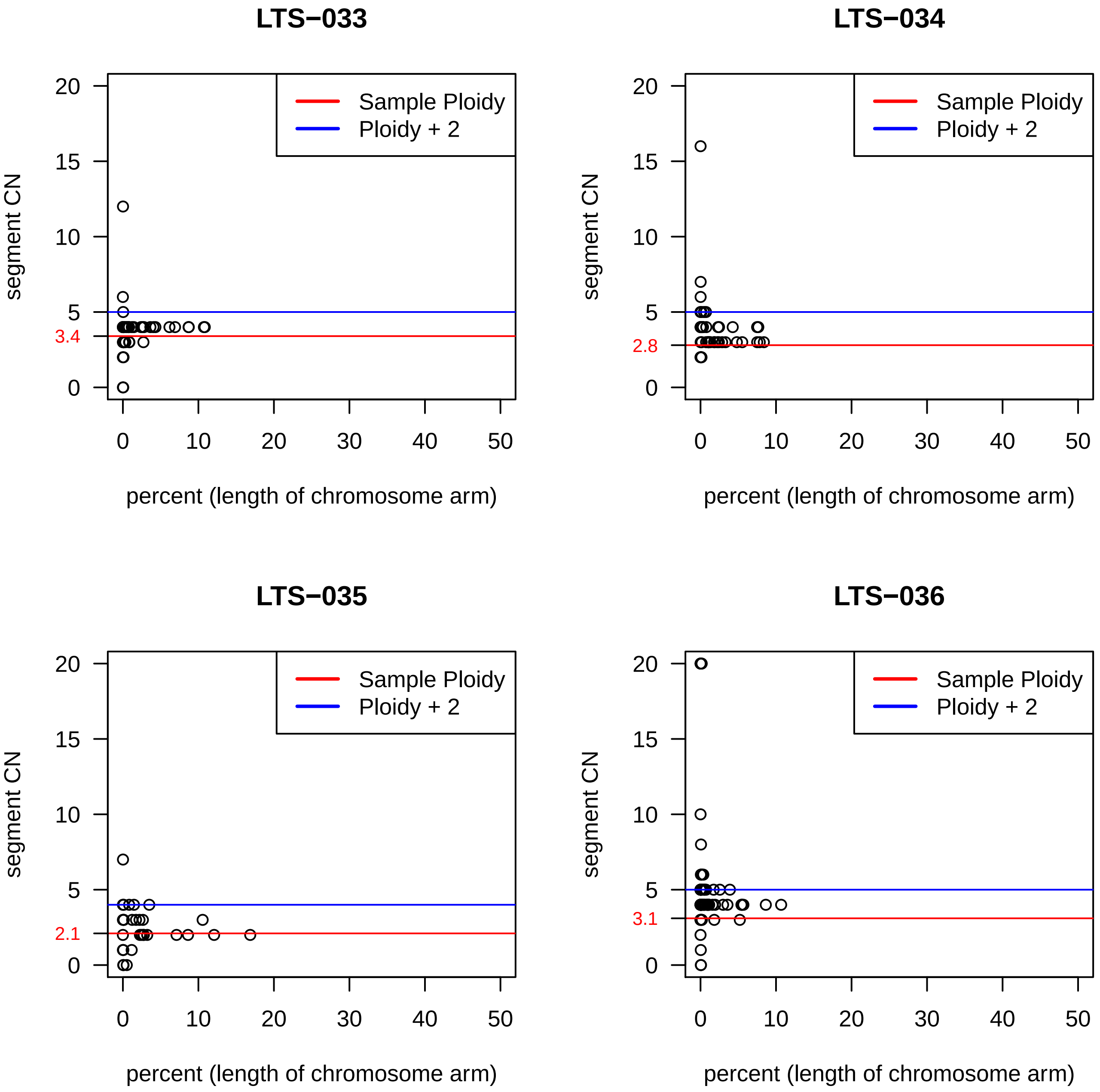

LTS−033
Sample Ploidy
Ploidy + 2
segment CN
3.4
percent (length of chromosome arm)
LTS−034
Sample Ploidy
Ploidy + 2
segment CN
2.8
percent (length of chromosome arm)
LTS−035
Sample Ploidy
Ploidy + 2
segment CN
2.1
percent (length of chromosome arm)
LTS−036
Sample Ploidy
Ploidy + 2
segment CN
3.1
percent (length of chromosome arm)

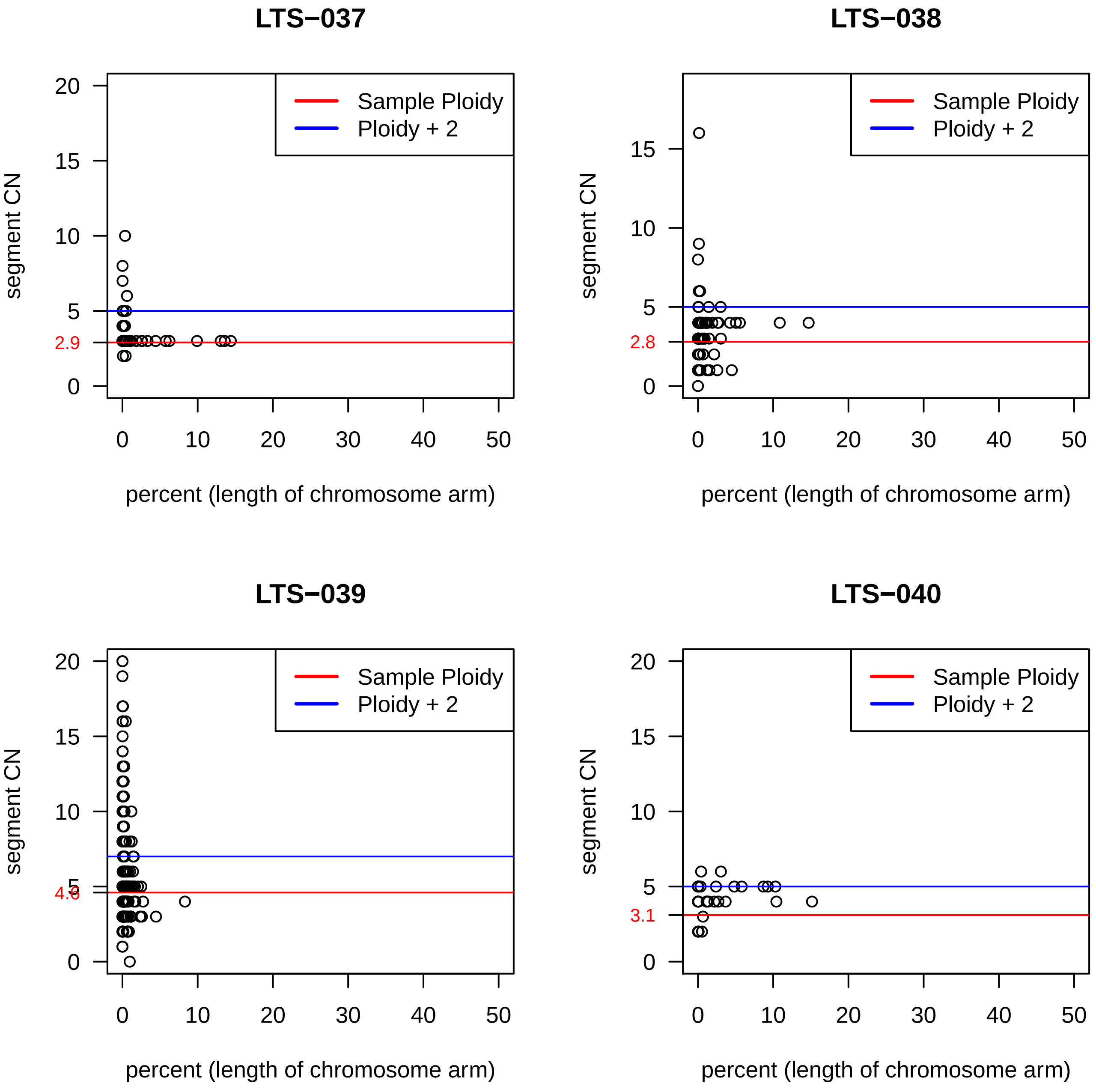
LTS−037
Sample Ploidy
Ploidy + 2
segment CN
2.9
percent (length of chromosome arm)
LTS−038
Sample Ploidy
Ploidy + 2
segment CN
2.8
percent (length of chromosome arm)
LTS−039
Sample Ploidy
Ploidy + 2
segment CN
4.6
percent (length of chromosome arm)
LTS−040
Sample Ploidy
Ploidy + 2
segment CN
3.1
percent (length of chromosome arm)

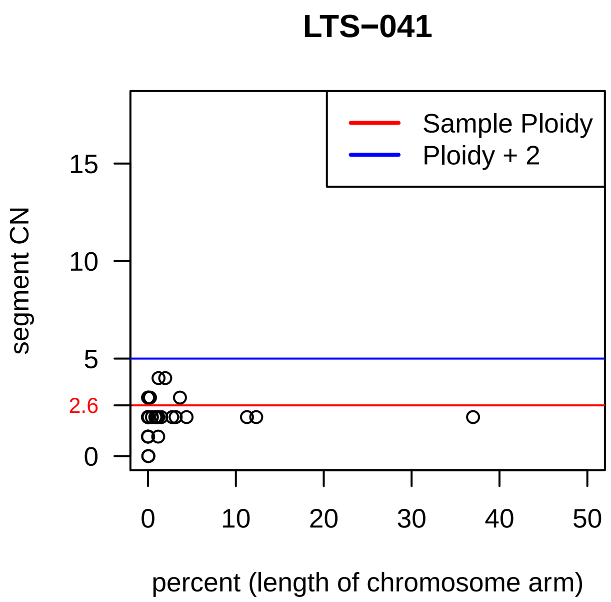
LTS−041
Sample Ploidy
Ploidy + 2
segment CN
15
10
5
2.6
0
0
10
20
30
40
50
percent (length of chromosome arm)
